# Immunomodulatory Response of Toll-like Receptor Ligand–Peptide Conjugates in Food Allergy

**DOI:** 10.1021/acschembio.1c00765

**Published:** 2021-11-11

**Authors:** Jorge Losada Méndez, Francisca Palomares, Francisca Gómez, Pedro Ramírez-López, Javier Ramos-Soriano, Maria Jose Torres, Cristobalina Mayorga, Javier Rojo

**Affiliations:** † Glycosystems Laboratory, Instituto de Investigaciones Químicas (IIQ), CSIC—Universidad de Sevilla, 41092 Seville, Spain; ‡ Allergy Unit, IBIMA, Regional University Hospital of Malaga, UMA, 29009 Malaga, Spain; § Allergy Clinical Unit, Hospital Regional Universitario de Málaga, 29009 Málaga, Spain; ∥ Nanostructures for Diagnosing and Treatment of Allergic Diseases Laboratory, Centro Andaluz de Nanomedicina y Biotecnología-BIONAND, 29590 Málaga, Spain; ∇ Allergy Research Group, Instituto de Investigación Biomédica de Málaga-IBIMA, 29009 Málaga, Spain; # Medicine Department, Universidad de Málaga-UMA, 29009 Málaga, Spain

## Abstract

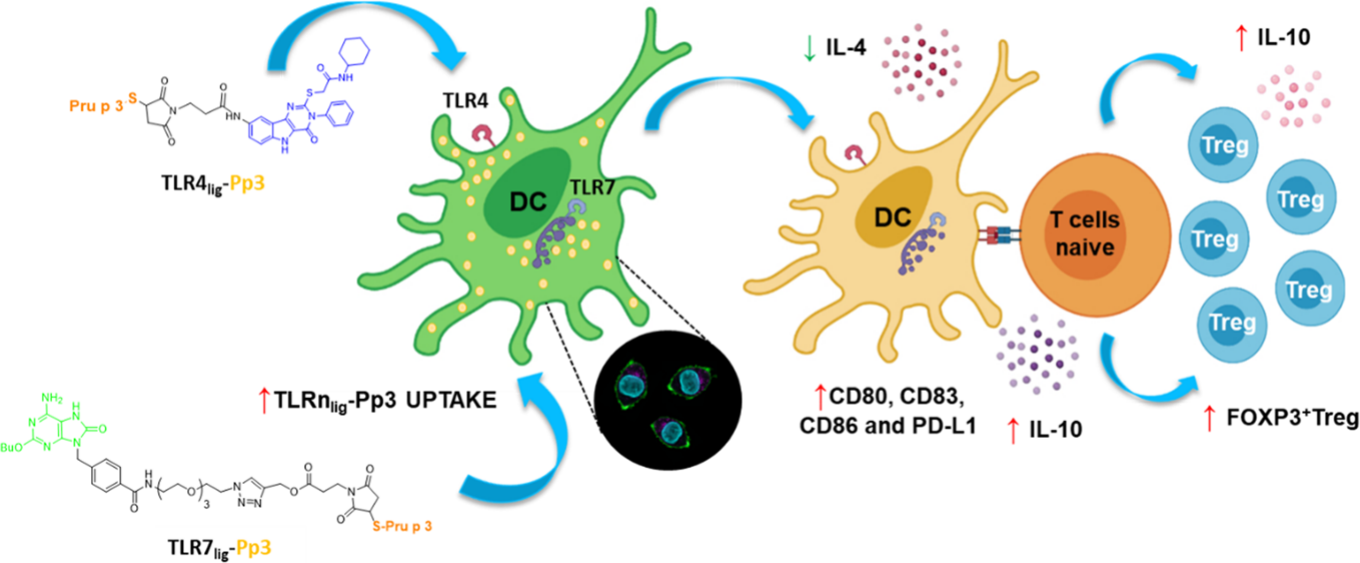

Covalent conjugation of allergens to toll-like receptor (TLR) agonists appears to be a powerful strategy for the development of safety compounds for allergen-specific immunomodulatory response toward tolerance in allergy. In this work, we have synthesized two family of ligands, an 8-oxoadenine derivative as a ligand for TLR7 and a pyrimido[5,4-*b*]indole as a ligand for TLR4, both conjugated with a T-cell peptide of Pru p 3 allergen, the lipid transfer protein (LTP) responsible for LTP-dependent food allergy. These conjugates interact with dendritic cells, inducing their specific maturation, T-cell proliferation, and cytokine production in peach allergic patients. Moreover, they increased the Treg-cell frequencies in these patients and could induce the IL-10 production. These outcomes were remarkable in the case of the TLR7 ligand conjugated with Pru p 3, opening the door for the potential application of these allergen–adjuvant systems in food allergy immunotherapy.

## Introduction

Food allergy (FA) is currently a burden for the Health Systems mainly in western European countries where the prevalence is increasing with plant origin as the main triggers in both adult and adolescent populations.^1^ Lipid transfer proteins (LTPs) are the main allergens related to plant FA in Mediterranean population although they are increasingly being observed in other European countries.^2,3^ Although some patients can selectively react to a single LTP (frequently Pru p 3, from peach), these proteins are considered panallergens, with patients sensitized to LTPs from different allergenic sources that can be taxonomically unrelated increasing the complexity of their clinical management.^4^ The high number of plant foods involved, independently of the severity of the reactions, has an important impact on the quality of life of patients because they will require very restrictive diets.

At present, allergen-specific immunotherapy (AIT) represents the unique approach able to modify the disease-inducing tolerance by the immunological modulation from the type 2 pattern to a type 1 or regulatory (Treg) response.^5,6^ However, conventional approaches using allergenic extracts have important drawbacks in terms of efficacy, safety, duration, and patient compliance.^7^ Therefore, novel vaccines that overcome such inconveniences are in demand.

The improvement of the efficacy would not only depend on the allergen but also on the adjuvants used in the vaccine composition.^8^ The main role of these adjuvants administered together with the allergens has shown to enhance the Th1 and Treg response by different mechanisms.^9^ Among the adjuvants, sequences containing inflammatory danger signals, such as “pathogen-associated molecular patterns” (PAMPs) present in microorganisms, can elicit the release of inflammatory cytokines and chemokines that initiate the defensive or the innate immune response.^10,11^ This effect is induced through the interaction with cellular receptors as pattern-recognition receptors^12^ that include a wide variety of families as C-type lectins (CLRs), toll-like receptors (TLRs), among others.^13^ TLRs are a type I transmembrane receptors involved at the beginning of the innate immune response.^14^ Ten functional TLRs (TLR1–10) are present in humans,^15^ each of them recognizing selectively different PAMPs, usually biomacromolecules. Some TLRs can be considered as very interesting candidates to produce a strong Th1 (nonallergic) response, counteracting the function and cytokine production of the Th2 cells from allergic patients.^16^ In this sense, studies performed with agonists of these TLRs as adjuvants have been explored in the framework of the design of new vaccines for immunotherapies for allergic diseases.^17−19^ From them, synthetic TLR4 agonists have progressed to be used in AIT.^20^ In this context, the use of the agonist for TLR4 monophosphoryl-lipid A (MPLA), a less toxic derivative of LPS, together with grass pollen allergens, has been shown to be a booster for AIT, inducing the IFNγ production and reducing the IgE levels in allergic patients.^21,22^ In addition, the conjugation of MPLA to ovalbumin (OVA) protein has been reported to promote dendritic cell (DC) maturation and induce a Th1 response.^15,23^ A further small-scale in vitro study in allergic patients has identified MPLA as potentiating allergoid responses in AIT.^24^ On the other hand, several TLR9 agonists have been assessed in combination with an allergen in clinical trials of AIT, demonstrating a strong capacity to induce Th1 response and consequently providing benefit when administered as an adjuvant to AIT.^25^ TLR9 activation has been shown to be capable of producing a Th1 response with IFNγ production and IgE synthesis inhibition using modified oligodeoxyribonucleotides containing CpG motifs in a FA animal model^9^ and by the co-administration of chenopodium album allergens and CpG in allergic rhinitis patients.^26,27^ Regarding TLR7 agonists, there are few studies indicating their potential used in AIT,^28,29^ despite imidazoquinoline compounds (imiquimod and resiquimod), TLR7 agonists have consistently demonstrated a capacity to reverse Th2 responses in favour of an anti-allergic Th1 response and IL-10 production.^30,31^ In addition, one study utilizing nanoparticles with an OVA peptide in the presence/absence of imidazoquinoline compound demonstrated the production of tolerogenic DCs and the induction of Tregs capable of suppressing the response to food challenge.^32^ Although TLR7 agonists are utilized in clinical settings,^29,33^ there are few studies highlighting their effects in modifying human food allergic responses.

Despite recent advances in the use of agonists in modulating the TLR4 or TLR7 to induce a Th1 response,^20^ these studies have certain limitations such as the short duration of the regimens studied, the level of allergen doses evaluated, or the low immunomodulatory profile of TLR agonists. Therefore, it is needed to optimize the adjuvanted-allergen immunotherapy regimens and to define immunological properties of TLR ligands, which act as agonists enhancing the tolerance response to food allergens.

Here, based on previous precedents and taking into consideration the synthetic accessibility of agonists to further functionalization, we have focused on TLR4 and TLR7 ligands (TLR4_lig_ and TLR7_lig_) to address our allergen conjugates with the aim to develop compounds for AIT in FA. We have addressed the immunological response induced by compounds that include, besides the TLR4 or TLR7 agonists, a synthetic peptide of the allergenic epitope Pru p 3 (Pp3), TLR4_lig_-Pp3, and TLR7_lig_-Pp3, respectively, in cells from LTP allergic patients and analyzed the type of response in order to assess the modulatory capacity of these ligands. Our findings indicate that this approach could be considered an interesting synthetic strategy for the development of new vaccines for FA immunotherapy.

## Results and Discussion

### Synthesis of the TLR4 Agonist (TLR4_lig_)

**Figure 1:**
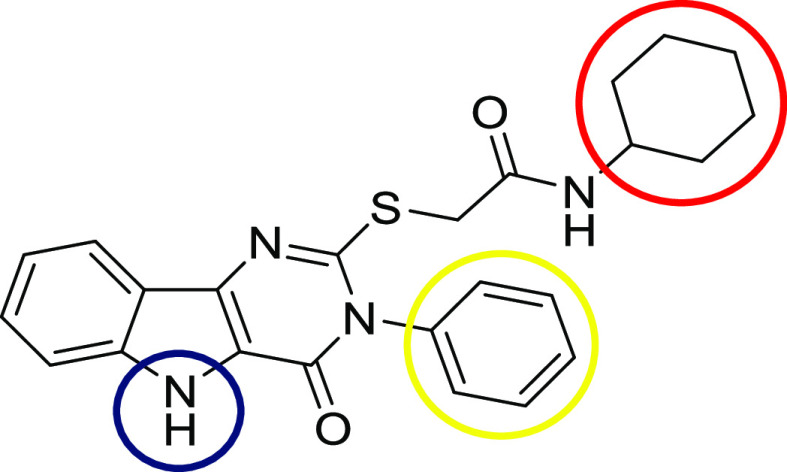
Pyrimido[5,4-*b*]indole derivative, a ligand for TLR4, and regions that influence the IFN type I promotion: the N-3 position (yellow region) must include a hydrophobic group, preferably an unsubstituted phenyl ring; the indole N-5 position (blue region) has to be unsubstituted; and the terminal amide (red region) required a highly hydrophobic substituent such as a cyclohexyl residue.

We have focused our efforts to prepare a TLR4 agonist based on pyrimido[5,4-*b*]indole derivatives. In the course of a high-throughput screening to identify activators of innate immunity,^34^ a series of pyrimido[5,4-*b*]indoles were recently discovered by Cottam as selective TLR4 agonists.^35^ In these studies, the fragments of these molecules that were implicated directly in the interaction with the TLR4 receptor were identified (Figure 1).

**Scheme 1:**
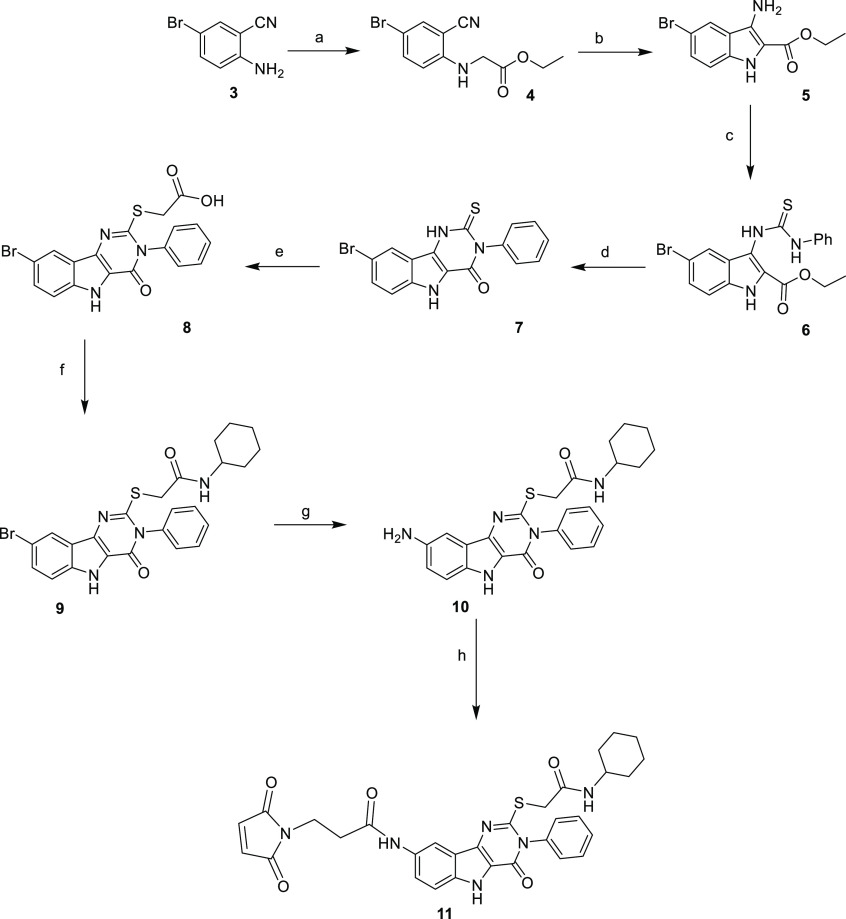
Synthesis of Pyrimido[5,4-*b*]indole Derivative **11** Reagents and conditions: (a) ethylbromoacetate, K_2_CO_3_, DMF, 75 °C, (63%); (b) KO^*t*^Bu, THF, <30 °C (39%); (c) PhNCS, EtOH, reflux (93%); (d) acetyl chloride, EtOH, reflux (72%); (e) BrCH_2_COOH, KOH, EtOH, rt (quant.); (f) cyclohexylamine, HATU, TEA, DMF, rt (quant.); (g) CuI, NaN_3_, NaAsc, DMEN, DMSO/H_2_O, 90 °C (72%); (h) 3-maleimidopropionic acid, HATU, DIPEA, DMF, rt (73%).

From these studies, the benzyl ring of the pyrimido[5,4-*b*]indole appeared as the most convenient site for structure modification without altering the binding properties of the molecule, a fact confirmed later on by the same group.^36^ Considering this precedent, we decided to modify the C8 position of the pyrimido[5,4-*b*]indole ring for the introduction of a maleimide group, required for the peptide (allergen) conjugation via a thiol–ene reaction (compound **11**, Scheme 1). We carried out the synthetic strategy depicted in Scheme 1, using the C8 position of the pyrimido[5,4-*b*]indole conveniently derivatized.

Briefly, compound **3** was synthesized by brominating the commercially available 2-aminobenzonitrile with NBS and acetonitrile as described in the literature.^37^ Then, the N-alkylation reaction to obtain compound **4** was optimized using more energetic conditions (heating at 75 °C in DMF) and with an excess of ethylbromoacetate (6 equiv), the yield being notably improved (up to 63%). Intermediates **5–7** were synthesized according to the literature procedures.^36^ Then, the formation of the thioether **8** with the free carboxylic acid by reaction of **7** with bromoacetic acid was directly achieved in quantitative yield. The next step consisted of the amidation reaction of the carboxylic acid **8** with cyclohexylamine using HATU as coupling agent, yielding amide **9** in high yield. Compound **9** was submitted to an Ullmann-type coupling, catalyzed by Cu(I), providing the aniline derivative **10**. The last step of the synthetic route was the introduction of a maleimide group using 3-maleimidopropionic acid,^38^ with HATU as the coupling agent, to give the maleimide derivative **11**, ready to be conjugated with the selected allergen.

### Synthesis of the TLR7 Agonist (TLR7_lig_)

**Figure 2:**
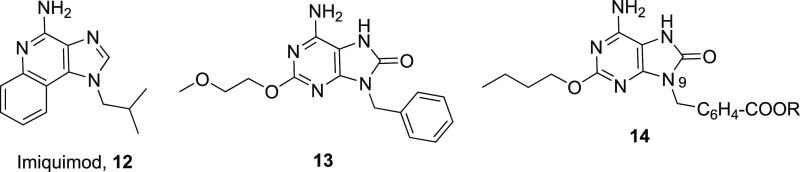
Structure of TLR7 ligands described in the literature.

For TLR7, there were some examples of small molecules developed as agonists. In 2002, it was shown that imidazoquinolines were capable of activating TLR7;^39^ however, it was not until 2004 that the identification of the natural ligand for TLR7, guanine and uridine-rich single-stranded RNAs, was reported.^40^ Within the family of imidazoquinolines (Figure 2), Imiquimod (**12**) is a drug approved for external genital warts caused by human papillomavirus infection.^41^ Adenine derivatives were identified as IFN inductors.^42^ Later, 9-benzyl-2-(2-methoxyethoxy)-8-oxoadenine (**13**) was reported as a TLR7 agonist (Figure 2).^43,44^ Therefore, all these structures could be considered as candidates for allergy vaccine adjuvants due to their ability to modulate the Th1/Th2 immune response.

Recently, a TLR 7 agonist was conjugated with a peptide derived from OVA,^45^ showing that the conjugate promoted the DC maturation through the production of IL-12p40 and CD86, as well as the T-cell proliferation. Isobe’s group reported that some modifications in the N-9 position of the aryl fragment of compound **14** (Figure 2) had not any significant effects on their biological activity.^46^

**Scheme 2:**
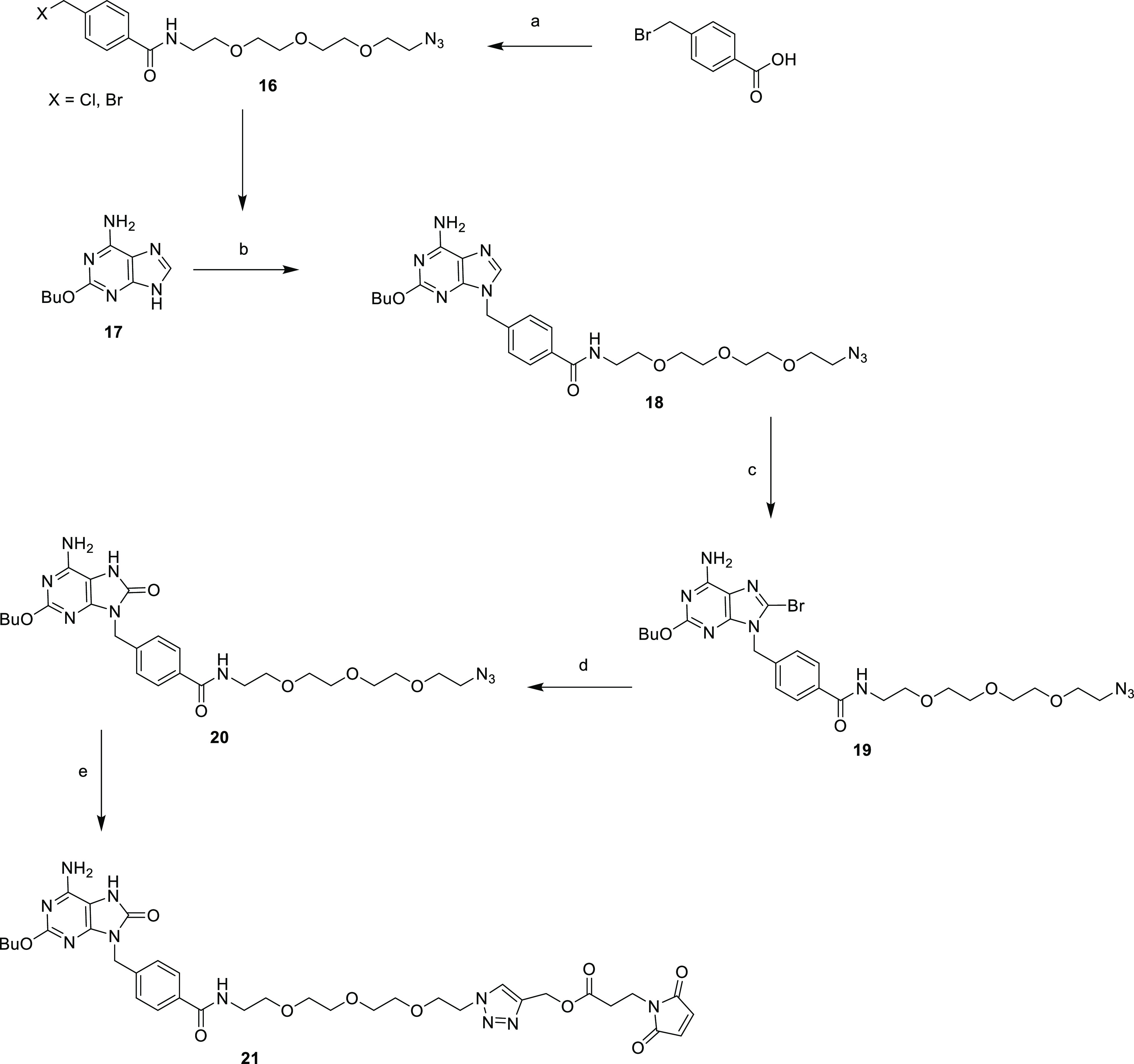
Synthesis of Adenine Derivative **21** Reagents and conditions: (a), SOCl_2_, 2-(2-(2-(2-azidoethoxy)ethoxy)ethoxy)ethan-1-amine (**15**), DCM, reflux (60%); (b) K_2_CO_3_, DMF, 65 °C (69%); (c) Br_2_, CHCl_3_, rt (66%); (d) HCO_2_H, reflux (47%); (e) prop-2-yn-1-yl-3-maleimidopropanoate, CuBr, TentaGel-TBTA resin, ACN/DMSO, rt (94%).

We were inspired by these precedents concerning small ligands as agonists for TLR7, and our synthetic strategy was focused on the introduction of a TEG-based linker in the benzyl residue at the N-9 position of compound **14** to conjugate the allergen (Scheme 2). This spacer should provide enough separation between the ligand and the allergen to avoid interferences during the binding to the TLR7 receptor through the adenine moiety.

The synthetic route to synthesize compound **21** is depicted in Scheme 2. 4-Bromomethylbenzoic acid was treated with thionyl chloride to obtain the corresponding acyl chloride, which was immediately reacted with the amino linker **15**,^47^ under anhydrous conditions, yielding the amide **16**. This intermediate was obtained as a mixture of Cl- and Br-derivatives in the benzylic methylene due to undesired partial chlorination by thionyl chloride treatment; however, this did not interfere with the synthetic pathway. The next step was the N-alkylation of 2-butoxy-9*H*-purin-6-amine **17**, synthesized according to the literature,^46^ with **16** in the presence of potassium carbonate to obtain compound **18** in 69% yield. Then, the electrophilic aromatic substitution with elemental bromine in chloroform provided the 8-brominated derivative **19**. Bromine at position C-8 was hydrolyzed under high-energy conditions with refluxing formic acid to give compound **20** in moderate yield. Finally, this compound was conjugated with prop-2-yn-1-yl-3-maleimidopropanoate,^18^ via Cu(I) catalyzed azide–alkyne 1,3 dipolar cycloaddition (CuAAC) to provide the maleimide derivative **21** in excellent yield.

### TLR7 and TLR4 Allergen Conjugation (TLR7_lig_-Pp3 and TLR4_lig_-Pp3)

Once TLR4 and TLR7 ligands **11** and **21**, respectively, were prepared conveniently functionalized with a maleimide group, we afforded the conjugation of the allergen to these adjuvants. The allergen (Pp3),^48^ whose sequence is SSNGIRNVNNLARTPDRQAC, was based on the region 26–46 of the Pru p 3 protein with an additional terminal cysteine. This peptide, conjugated with a glycodendrimer, was previously tested using in vitro and in vivo experiments to induce tolerance against peach allergy.^18^

**Figure 3:**
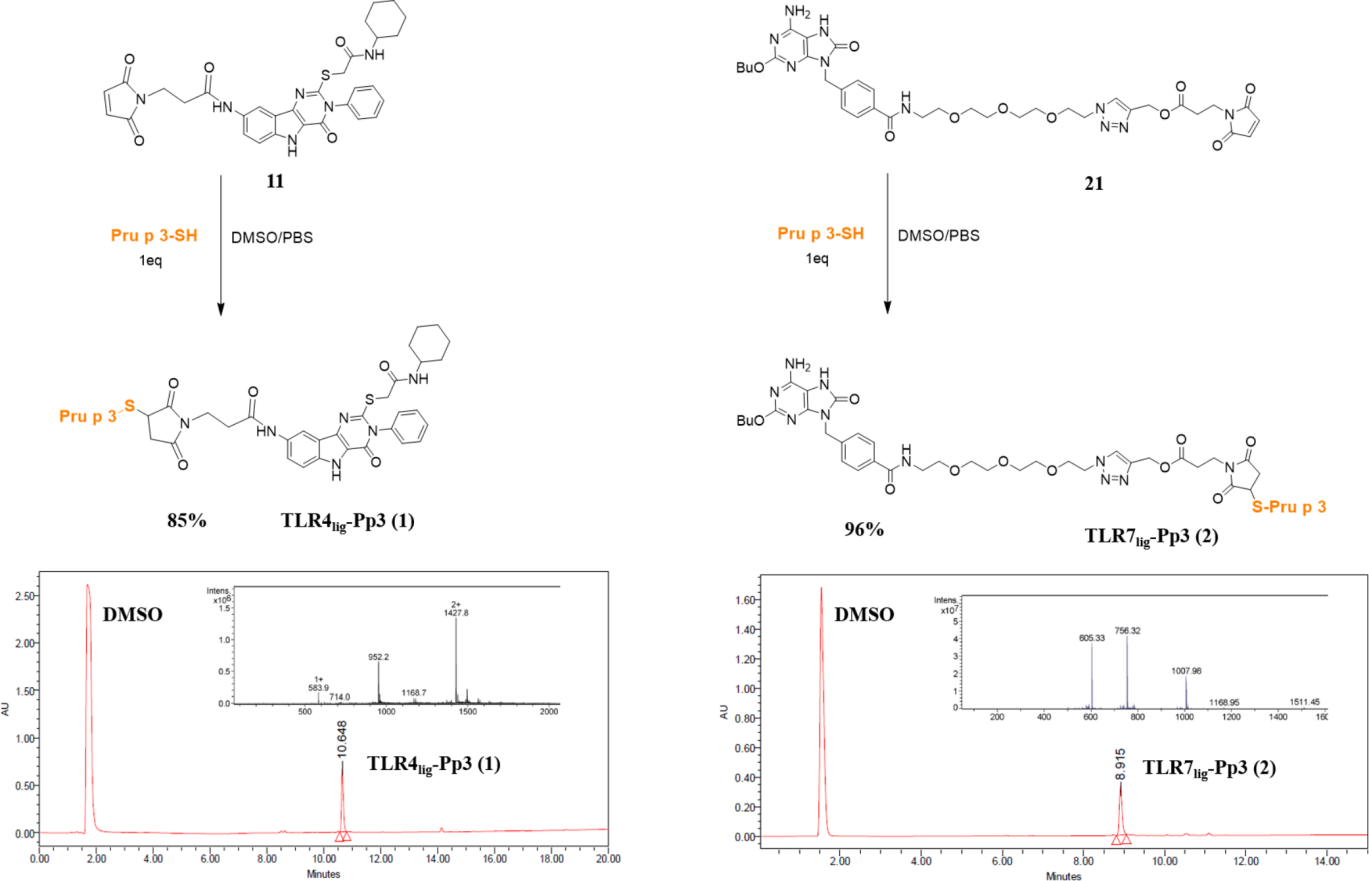
Conjugation of Pp3 to maleimide scaffolds **11** and **21** to obtain the conjugates TLR4_lig_-Pp3 (**1**) and TLR7_lig_-Pp3 (**2**), respectively (top) and their corresponding ESI-MS spectra and HPLC chromatograms (bottom).

The covalent conjugation of Pp3 to **11** and **21** was achieved through the click thiol–ene reaction. The conditions for this conjugation were optimized to obtain the corresponding conjugates directly in 30 min (monitored by RP-HPLC) combining the maleimide derivative and the peptide in an equimolar ratio in dimethylsulfoxide (DMSO)/phosphate buffered saline (PBS). The corresponding conjugates TLR4_lig_-Pp3 (**1**) and TLR7_lig_-Pp3 (**2**) were obtained in high yield and purity. These conjugates were successfully characterized by MS spectrometry (Figure 3).

### TLRn_lig_-Pp3 Uptake

**Figure 4:**
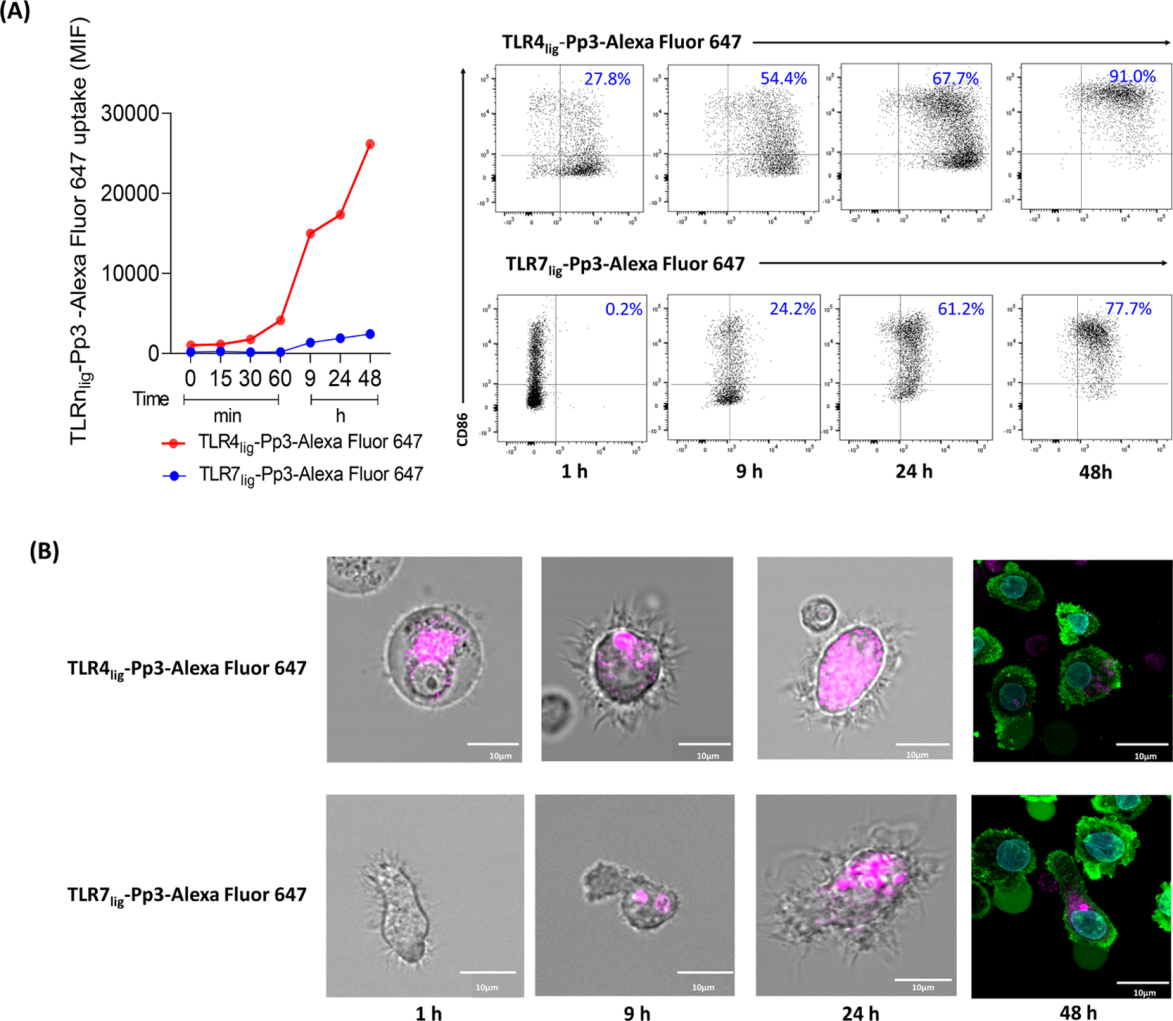
TLRn_lig_-Pp3 internalization and localization. (A) Timing of fluorescence-labeled TLRn_lig_-Pp3 uptake by moDCs and flow cytometry plots at different time points, showing the TLRn_lig_-Pp3 percentages on CD86^+^ moDCs. (B) Representative confocal images of moDCs incubated with TLRn_lig_-Pp3 at 10 nM at different time points (from 1 to 48 h). Data are consistent with the flow cytometry analysis showing a high fluorescence signal (pink) from TLR4_lig_-Pp3 at 1 h and TLR7_lig_-Pp3 at 9 h inside the moDCs. The confocal images at 48 h show different cellular regions. The sub-membrane actin was stained with Atto 488-phalloidin (green) and nuclei with Hoechst (blue).

We have analyzed the level of uptake of TLRn_lig_-Pp3 on monocyte-derived DCs (moDCs), using 10 nM of TLR4_lig_-Pp3 (**1**) or TLR7_lig_-Pp3 (**2**), both labeled with Alexa fluor 647, by flow cytometry and confocal microscopy (CM).^49^ Flow cytometry measurements indicated that TLRn_lig_-Pp3 were uptaken by moDCs in a time-dependent manner, which is faster and higher for TLR4_lig_-Pp3 compared to TLR7_lig_-Pp3 (Figure 4A). Furthermore, the dot plots and CM images showed that the internalization of the TLR4_lig_-Pp3 was visible from the first hour of incubation, while for the TLR7_lig_-Pp3, it appeared after 9 h (Figure 4A,B). The moDC uptake continued to increase after 48 h for both TLRn_lig_-Pp3, achieving similar percentages of positive cells. Additionally, the CM images indicate that TLRn_lig_-Pp3 intracellular distribution in moDCs was different for each ligand, with TLR7_lig_-Pp3 showing perinuclear accumulation, while with TLR4_lig_-Pp3, an accumulation nearest to membrane and cytoplasm was observed (Figure 4B).

These differences in the internalization time and in the location of the TLRn_lig_-Pp3 in the moDCs can be due to the differential position of their receptors because the expression of TLR4 is at the cell surface, while TLR7 is at intracellular compartments, such as endosomes.^50^ These internalization pathways would facilitate food allergen uptake to increase the effective response. Therefore, this TLRn_lig_-Pp3 could contribute to the design of efficacious, shorter, and safer immunotherapy protocols.^51^

### TLRn_lig_-Pp3 Induce Changes in the moDC Maturational Status and Cytokines Production

**Figure 5:**
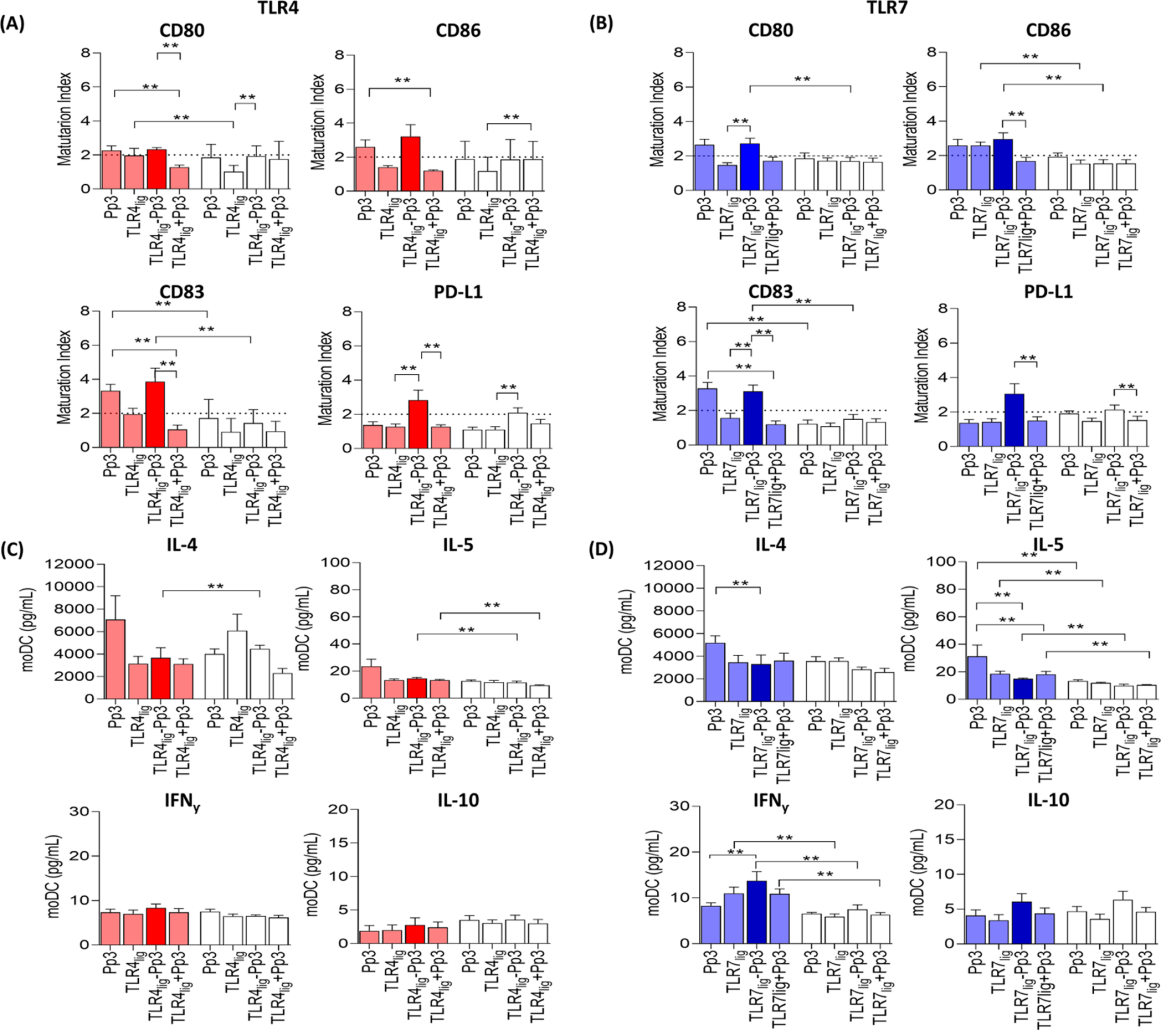
TLRn_lig_-Pp3 change moDC maturation and cytokine production. Bars represent median and SEM of the (A,B) MI for the different surface markers on moDCs and of the (C,D) cytokine production in supernatants from moDCs assays for Pp3, TLRn_lig_, TLRn_lig_-Pp3, and TLRn_lig_ plus Pp3 for allergic patients (*n* = 9, color bars) and tolerant controls (*n* = 9, white bars) at 10 nM. The Mann–Whitney *U* test was used for pairwise comparisons between unrelated groups and Wilcoxon signed-rank test was used for pairwise comparisons in related samples, showing significant differences as **, (*p* < 0.0125 and *p* < 0.0010, Bonferroni correction, respectively). The dotted line represents the MI > 2.

During the first steps of the immune response, the antigen interacts with moDCs inducing their maturation. The type of maturation response that will be characterized by the expression of co-stimulatory molecules and pattern of cytokine production will orchestrate the further immunological response mediated by T helper lymphocytes.^52^ The assessment of the TLRn_lig_-Pp3-induced maturational phenotypical changes in the expression of activation/regulation (CD83 and PD-L1) and maturation (CD80 and CD86) cell surface markers indicated that compared to tolerant controls, there was a significant increase of CD80, CD83, and CD86 in allergic patients when moDCs were stimulated with TLR7_lig_-Pp3, while only of CD83 with TLR4_lig_-Pp3 (Figure 5A,B). The analysis of the effect of the structures including the TLR4_lig_ (**10**) and TLR7_lig_ (**20**) ligands but not Pp3 indicated that were also able to stimulate CD80 and CD86, respectively, in allergic patients compared to tolerant controls. This effect is in agreement with previous studies,^53^ in which it was shown that different TLR adjuvants (without allergen) were able to modulate the moDC activity/maturation in birch pollen allergic patients and animal model.^53^ This capacity to activate the innate response, enhancing the moDC immunomodulation, has also been described for other small synthetic TLR4 and TLR7 ligands.^54,55^

These data suggest that the changes in the expression of CD83 induced by TLRn_lig_-Pp3 are related to the specific response generated by the allergen peptide because we have found differences not only between allergic patients and tolerant controls but also between the structures with (TLRn_lig_-Pp3) and without (TLRn_lig_) peptide. This confirms the results of previous reports where it has been described that TLR ligands can show differential expression between the co-stimulatory molecules, high CD83 expression, and a slightly reduced CD80 and CD86 expression.^56^

We have analyzed in allergic patients the effect of the insertion of the Pp3 peptide conjugated to TLR ligands (TLRn_lig_-Pp3) compared to the effect of including these elements in a separated way. Data showed that TLR4_lig_-Pp3 led to a significant increase of CD80, CD83, and PD-L1 compared to the stimulation with TLR4_lig_ plus Pp3 peptide in a separate way (Figure 5A). Furthermore, for TLR7_lig_-Pp3, we observed a significant increase of CD83, CD86, and PD-L1 compared to TLR7_lig_ plus Pp3 peptide in a separate way (Figure 5B). These results showed that a more efficient immunological response is produced when both allergen and adjuvant interact together with the antigen-presenting cells (APC) as DCs. TLRn_lig_-Pp3 uptake correlates with functional changes in moDCs in vitro.^57,58^ Therefore, an efficient antigen presentation requires the activation of co-stimulatory molecules.^59^ Here, TLRn_lig_-Pp3 activate, regulate, and maturate the surface marker expression from moDCs, suggesting that the contact with TLRs is important for initiation the immune responses.

To further characterize the response pattern during the moDC maturation following incubation with TLRn_lig_-Pp3, we measured the cytokine release into the culture supernatant. Our analysis in allergic patients compared to tolerant controls detected significant increases of IL-5 and IFNγ with all the conditions containing TLR7_lig_ (TLR7_lig_, TLR7_lig_-Pp3, and TLR7_lig_ plus Pp3 in a separate way), indicating that this effect is rather due to TLR_lig_ (Figure 5D). In the case of TLR4 (Figure 5C), only significant increases of IL-5 were observed in allergic patients compared to tolerant controls when stimulating with TLR4_lig_-Pp3 and TLR4_lig_ plus Pp3 peptide in a separate way. No differences in the induction of IL-10 production were observed in allergic patients compared to tolerant controls for any of the ligands assayed (Figure 5D).

No differences were observed in the cytokine production after stimulation with TLRn_lig_-Pp3 and TLRn_lig_ plus Pp3 in a separate way. Interestingly, we observed that TLR7_lig_-Pp3 induced significant lower IL-4 and IL-5 levels, while higher IFNγ levels compared to the Pp3 only in allergic patients, indicating the specific immunomodulatory capacity of this compound.

Altogether, these data indicated that TLR7_lig_-Pp3 stimulation induced moDCs maturation accompanied by a decrease of Th2 pattern (IL-4 and IL-5) and an increase of the Th1 cytokine (IFNγ production).^20,51,60^ In fact, the interaction of a TLR7 ligand with its receptor triggers the IFNγ induction pathway as it has been described previously.^61^ Therefore, the effect of TLR7_lig_-Pp3 in the production of type 1 immune response is essential for the immunity and prevention of the development of Th2 responses,^62^ making it an attractive compound for AIT.

Regarding TLR4_lig_-Pp3, it promoted a moDC differential maturation in allergic patients with a reduction of Th2 cytokines compared to the peptide, although without changes in the IFNγ and IL-10 levels. This slight response in the production of cytokines (IFNγ and IL-10) could be associated with the maturation stage of the moDCs because the TLR ligands can influence in a different way the moDC activation, antigen presentation, co-stimulation, and cytokine production.^53,63^

### TLRn_lig_-Pp3 Stimulate the Specific Proliferative Response with a Th1 Profile

Our findings that the TLRn_lig_-Pp3 were uptaken by moDCs, affecting their activation and maturation, suggested that they could be involved in the following steps of the immunological response, where mature moDCs present the allergen to Th lymphocytes. To examine this possibility, we determined lymphocyte proliferation using homologous pre-stimulated moDCs as APCs and the profile of the response in terms of cytokine production. Different T-lymphocytes (CD3^+^T-, CD4^+^T-, and CD8^+^T-cells), B-cells (CD19^+^), and NK-cells (CD56^+^) subpopulations have been reported to shape immunological responses in different ways.

**Figure 6:**
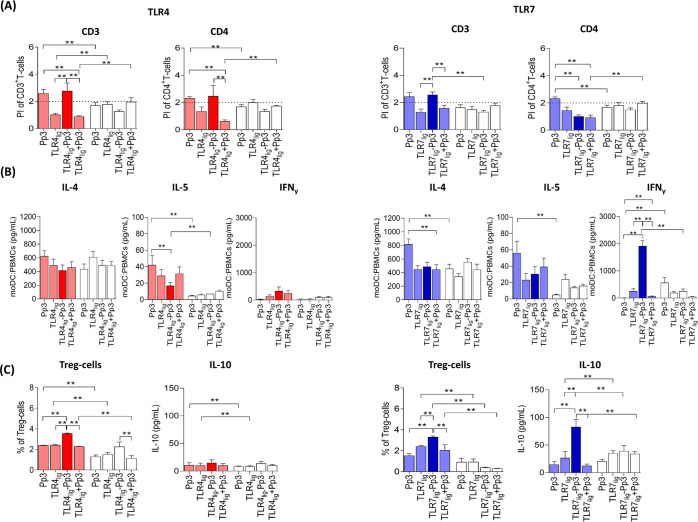
Specific lymphocyte proliferation and regulatory response in the presence of both TLRn_lig_-Pp3. Bars represent median and SEM of the (A) PI of T-cells, (B) of the cytokine production in supernatants from lymphocyte proliferation assay, and (C) of the Treg-cell percentages and IL-10 production for Pp3, TLRn_lig_, TLRn_lig_-Pp3, and TLRn_lig_ plus Pp3 for allergic patients (*n* = 9, color bars) and tolerant controls (*n* = 9, white bars) at 10 nM. The Mann–Whitney *U* test was used for pairwise comparisons between unrelated groups, and Wilcoxon signed-rank test was used for pairwise comparisons in related samples, showing significant differences as ** (*p* < 0.0125 and *p* < 0.0010, Bonferroni correction, respectively). The dotted line represents the PI > 2.

The results indicated that only TLR7_lig_-Pp3 led to significantly increased CD3^+^T-cell proliferation in allergic patients compared to tolerant controls, while TLR4_lig_-Pp3 seemed to stimulate the T-cell proliferation without inducing a significant difference between groups (Figure 6A). No difference in the proliferative response was found for other subpopulations as B-cells and NK-cells with any of the TLRn_lig_-Pp3 between allergic patients and tolerant controls because the proliferation of that cell subpopulation was negative [proliferation index (PI) < 2] (Figure S1).

Moreover, in allergic patients, there was a significant increase in CD3^+^ and CD4^+^ T-cell proliferation after stimulation with TLR4_lig_-Pp3 versus TLR_lig_ and versus TLR4_lig_ plus Pp3 peptide; additionally, TLR7_lig_-Pp3 induced a specific proliferative response of CD3^+^T-cells compared to TLR7_lig_ and TLR7_lig_ plus Pp3 peptide (Figure 6A). These results suggest that the TLRn_lig_-Pp3 induce a Pp3-specific proliferative response of T-lymphocytes.

Regarding the cytokine profile produced during the proliferative response, the most interesting results indicated that both TLRn_lig_-Pp3 induced a decrease of IL-4 and IL-5 levels compared to Pp3 peptide (alone), which is significant for IL-5 with TLR4_lig_-Pp3, and an increase of IFNγ is significant only for TLR7_lig_-Pp3 in allergic patients (Figure 6B). These changes that are observed only in allergic patients indicated that the compounds combining the allergenic peptide and the adjuvant had the capacity to modulate the Th2 response in a specific way.

### TLRn_lig_-Pp_3_ Stimulate the Treg Cells and IL-10 Production

To further investigate the capacity of TLRn_lig_-Pp3-treated moDCs to induce Treg cells, we performed co-culture experiments and analyzed the Treg cell percentages. The results indicated significantly higher percentages of Treg cells and IL-10 production in allergic patients compared to tolerant controls when stimulated with both TLRn_lig_-Pp3, being significant for TLR7_lig_-Pp3 (Figure 6C). Moreover, these effects were significantly higher compared to Pp3 peptide alone, TLRn_lig_ as well as TLRn_lig_ plus Pp3 only in allergic patients except with TLR4_lig_-Pp3 for the IL-10 production (Figure 6C). Regarding the TLR4 activation, despite producing IFNγ, they can induce a low production of IL-10, as has been observed in our experimental setup.^53^ In contrast, regarding TLR7 activation, it has been described in allergic animal models that using small molecular weight compounds, such as TLR7 ligands, the allergen-induced Th2 responses as well as the airway inflammation and airway hyper-reactivity can be suppressed through IL-10 secretion.^64,65^

These data suggested that TLRn_lig_-Pp3-activated moDCs promoted the presentation of the allergen (Pp3) and generated a specific immunological response with a type 1/Treg immunological pattern and the suppression of Th2 effector cells.

### Effect of the Combination of Both TLRn_lig_-Pp3 on Immunological Cells

After these results, we wonder whether the immunological effect would be enhanced by stimulating cells simultaneously with both TLRs, looking for a synergistic effect. It is known that the use of synthetic TLR4 and TLR7 ligands as adjuvants for vaccine in infection diseases induced a rapid, sustained, and broadly protective responses.^54^ Moreover, in a study in amoxicillin-induced maculopapular exanthema, it was shown that the simultaneous stimulation with TLR2 and TLR4 agonists could be critical for the induction of the specific immune responses, increasing the moDC maturation and T-cell proliferation and emulating the immune response the allergic patients had at the acute phase of the reaction after the drug administration.^66^ Recently, the dual role of TLR ligands in the allergic airway inflammation response in human and animal models has been described,^67^ influencing positively or negatively in the allergic response. In fact, it was described that TLR4 activation can mediate allergic responses.^68^

**Figure 7:**
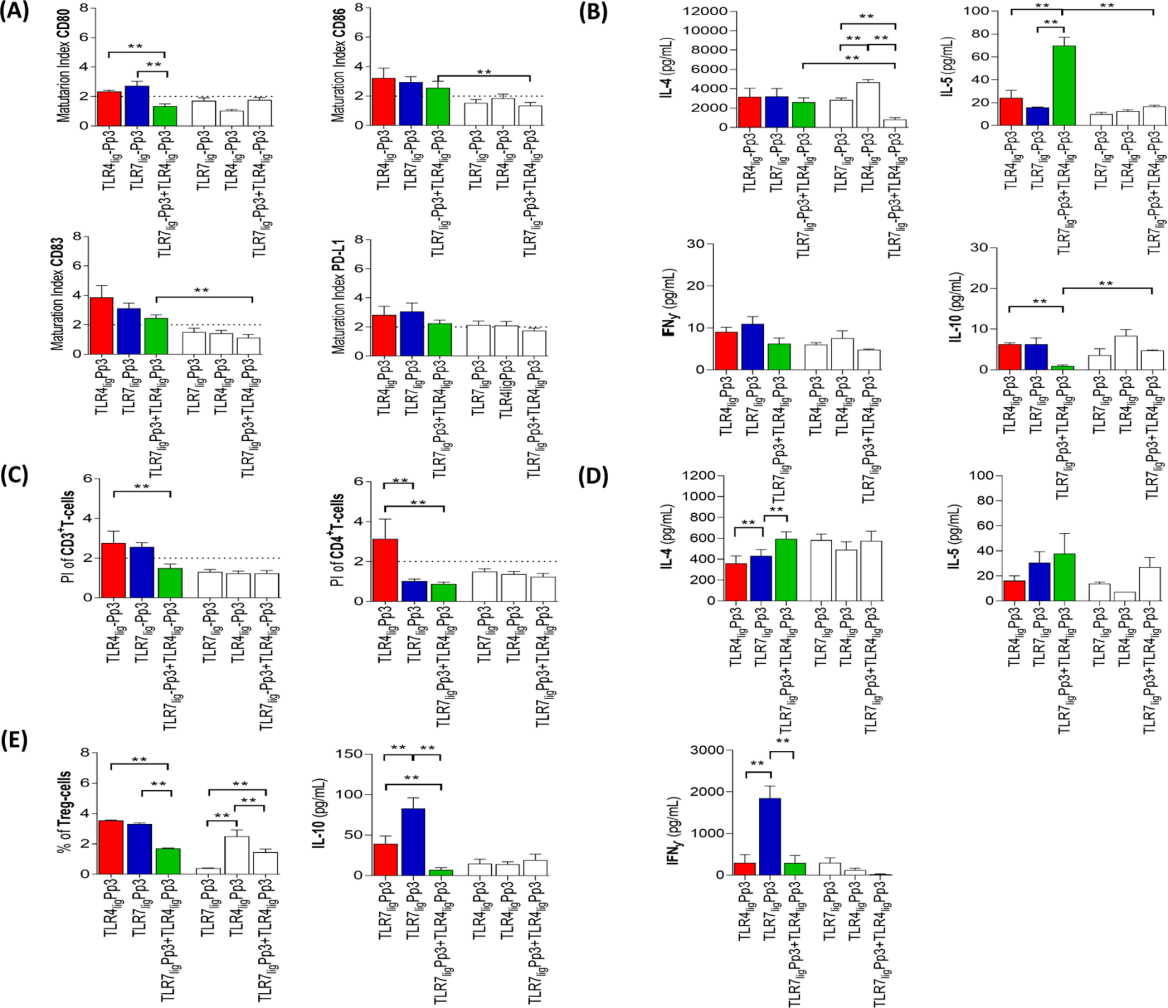
Effect of the combination of both TLRn_lig_-Pp3 on immunological cells. Bars represent median and SEM of the (A) MI for the different surface markers on moDCs, of the (B) cytokine production in supernatants from moDCs assays, of the (C) PI of T-cells, of the (D) cytokine production in supernatants from lymphocyte proliferation assay, and of the (E) Treg-cell percentages and IL-10 production for combination of both TLRn_lig_-Pp3 in allergic patients (*n* = 9, color bars) and tolerant controls (*n* = 9, white bars) at 10 nM. The Mann–Whitney *U* test was used for pairwise comparisons between unrelated groups, and Wilcoxon signed-rank test was used for pairwise comparisons in related samples, showing significant differences as ** (*p* < 0.016, Bonferroni correction, respectively). The dotted line represents the MI and the PI > 2, respectively.

However, our results indicated an opposite result because the combination of both TLRn_lig_-Pp3 (TLR7_lig_-Pp3 plus TLR4_lig_-Pp3) together decreased the maturation already produced by each compound alone, being significant for CD80, with an increase of IL-5 production and decrease of IL-10 only in allergic patients (Figure 7A,B). There was also a significant decrease of IL-4 production in tolerant controls. The rest of the combinations between the TLRn_lig_ (with or without Pp3 peptide) did not show any effect on the moDCs from allergic patients and tolerant controls (Figure S2A).

Regarding the effect of these interactions with moDCs on the proliferative response of the T-, B-, and NK-cells (Figure 7C), they were in line with the DC maturation results because there was also a decrease in comparison with the proliferation induced by the ligands incubated separately with a significant increase of IL-4 production and decrease of the IFNγ level (Figure 7C,D). In other words, the combination of both TLR ligands can attenuate the inflammatory response induced by the compounds alone associated with type 1 immunological profile (decrease of IL-4 and IL-5 and increase of IFNγ). Similarly, when we analyzed the regulatory pattern (Figure 7E), we observed a significant decrease of Treg percentages as well as the IL-10 production in response to the combination of TLR7_lig_-Pp3 plus TLR4_lig_-Pp3 compared to each TLRn_lig_-Pp3 in allergic patients.

All these results show that TLRn_lig_-Pp3 activated the immunological response by stimulation of TLR4 and TLR7, promoting the maturation of moDCs, through of a regulatory pattern with IFNγ and IL-10 production, while their combinations neutralize their individual effects in allergic patients. This indicates that TLRn_lig_-Pp3 (alone) improve their immunogenic characteristics compared to their combinations, suggesting that they could be used as a single component for FA vaccines.

## Conclusions

In the last years, the discovery of novel, safe, and effective forms of short-duration allergen immunotherapy for FA has attracted considerable scientific interest. In this work, we have developed a straightforward synthetic strategy to combine a TLR ligand with an allergen in a well-defined single structure to induce a regulatory response in FA. These compounds were tested successfully in in vitro assays using moDCs from allergic patients to LTPs.

We have provided evidence that TLRn_lig_ coupled to Pp3, TLRn_lig_-Pp3, are captured by moDCs and stimulate their maturation and activation, increasing the PD-L1 expression. This suggests that moDCs have the capacity to present Pru p 3 to immune cells promoting the induction of Treg cells in LTP allergic patients that will supress the effector response. These effects are significantly higher in the case of TLR7_lig_-Pp3.

The covalent conjugation of the allergen to the TLR ligand in a single entity is fundamental to improve the outcome of the adjuvant, increasing the stimulatory capacity in comparison with the noncovalent combination of the adjuvant (TLRn_lig_) together with the allergen (Pp3). However, we have not observed a synergistic effect when different TLRn_lig_ were combined in the experiments, being less productive that when they were applied separately.

This study sheds light into the basic structural and immunologic mechanisms that places TLRn_lig_ conjugated to Pru p 3 as novel suitable preparations to formulate improved vaccines for allergen immunotherapy.

## Methods

### Synthesis of Adjuvant–Allergen Conjugates

The synthesis and characterization of all new intermediates are described in detail in the Supporting Information.

### General Protocol for the Preparation of Allergen Conjugates (TLRn_lig_-Pp3)

A solution of peptide Pp3 (1.3 mg, 0.59 μmol) in H_2_O MQ (80 μL) and sodium phosphate buffer (50 mM, pH 7.4, 40 μL) was added to a solution of maleimide derivative **11** or **21** (0.59 μmol, 1 equiv) in DMSO (195 μL). After shaking for approx. 30 min at room temperature (the progression of the reaction was monitored by analytical RP-HPLC), only the signal of the corresponding final product was observed. Then, the reaction mixture was filtered (Ultra-15, MWCO 3 kDa) to remove salts and washed twice with H_2_O MQ. The supernatant was lyophilized to give the conjugated compounds TLR4_lig_-Pp3 (**1**) and TLR7_lig_-Pp3 (**2**).

### Allergic Patients and Tolerant Controls

This study included LTP-allergic patients and tolerant controls. Inclusion criteria were clear clinical history of peach allergy, a positive skin prick test (SPT), and specific IgE (sIgE) to Pru p 3 or a positive oral food challenge (OFC) to unpeeled-peach. OFC was not performed in patients with a history of more than two episodes of anaphylaxis after peach ingestion in the two years preceding the study. A tolerant control group of subjects with negative SPT and sIgE results and good tolerance to unpeeled peach was also included (Table S1). The study was conducted at the Allergy Service and Research Laboratory of the Hospital Regional Universitario de Málaga-Instituto de Biomedicina de Málaga (HRUM-IBIMA), Spain, in accordance with the Declaration of Helsinki. The study was approved by the local ethics committee. All participants signed informed consent forms before the study began.

### Generation of moDCs

Fresh peripheral blood mononuclear cells, obtained by a ficoll gradient, from 40 mL of blood per individual, were used for monocyte purification by means of anti-CD14 microbeads following the manufacturer’s protocol (Miltenyi Biotec, Bergisch Gladbach, Germany). The CD14– cell fraction was placed in 10% DMSO and frozen for a later lymphocyte proliferation test. To generate DCs, monocytes (CD14+ cells) were incubated in complete medium containing Roswell Park Memorial Institute 1640 medium (Thermo Fisher Scientific, Carlslab, CA) supplemented with 10% fetal bovine serum (Thermo Fisher Scientific), streptomycin (100 μg/mL), and gentamicin (1.25 U/mL), as well as recombinant human rhGM-CSF (200 ng/mL) and rhIL-4 (100 ng/mL) (both from R&D Systems Inc., Mineapolis, MN) for 5 days at 37 °C and 5% CO_2_.

### Fluorescent Labeling of TLR_lig_-Pp3

TLR7_lig_- and TLR4_lig_-Pp3 were labeled with Alexa Fluor 647 NHS Ester (succinimidyl ester) (TLR7_lig_- and TLR4_lig_-Pp3-Alexa Fluor 647) according to the manufacturer’s instructions (Thermo Fisher Scientific). After 1 h of incubation with constant stirring in the dark, the uncoupled free Alexa Fluor 647 was removed by gel filtration column (PD MiniTRap G-25 column, GE Healthcare, Chicago, IL) with PBS. The TLR7_lig_- and TLR4_lig_-Pp3-Alexa Fluor 647 were concentrated with a Nanodrop 2000 spectrophotometer (Thermo Fisher Scientific).

### TLR_lig_-Pp3 Uptake by Flow Cytometry and Confocal Microscopy

Immature moDCs were cultured with 10 nM TLR7_lig_- and TLR4_lig_-Pp3-Alexa Fluor 647 at different time points (from 15 min to 48 h) at 37 °C. The moDCs were stained with anti-human-CD86 [Becton Dickinson (BD) Bergen, NJ] and analyzed using flow cytometry. Cells were acquired in a FACSCanto II Cytometer (BD), and data were analyzed using FlowJo software (Tree Star, Inc., Ashland, OR). Results were expressed as mean intensity fluorescence and as percentages of double positive cells for CD86 and TLR7_lig_- and TLR4_lig_-Pp3-Alexa Fluor 647. Following incubation with TLR7_lig_-TLR4_lig_-Pp3 at different time points, moDCs were fixed in PBS containing 4% paraformaldehyde for 1 h, washed three times with PBS, stored, and protected from light at 4 °C until analysis. Sub-membrane actin and nuclei (DNA) were labeled by 20 min incubations with 10 μM Atto 488-conjugated phalloidin (Sigma-Aldrich, Munich, Germany) and 1 μg/mL Hoechst 33258 (Sigma-Aldrich), respectively. Once prepared, moDCs were either mounted on glass slides in Fluoroshield mounting medium (Sigma-Aldrich) or transferred to optical bottom 96-well plates (Nunc) in PBS for observation by CM. For the lysosome staining, after fixation, cells were permeabilized with saponin 0.1% in PBS with 2% bovine serum albumin V fraction (Sigma-Aldrich), followed by an overnight incubation at 4 °C with the primary rabbit LAMP-1 (Santa Cruz Biotechnology, Santa Cruz, CA) antibody using a 1:25 dilution followed by a 1 h incubation at room temperature with a secondary anti-rabbit Cy2-conjugated antibody (Jackson Laboratories, Bar Harbor, ME). Cells were washed three times after each antibody incubation and finally mounted on glass slides. Samples were analyzed using a Leica DM6000 inverted microscope connected to a Leica SP5 laser scanning confocal system and Fiji software.

### moDC Maturation Studies

To assess changes in the moDC maturation state after stimulation, 10^5^ moDCs/mL were incubated in 96-well plates (Thermo Fisher Scientific) with TLR4_lig_ and TLR7_lig_ (both ligands without Pru p 3), TLR ligands with Pru p 3 (TLR4_lig_-Pp3 or TRL7_lig_-Pp3), and Pru p 3 peptide (Pp3). In addition, different experimental combinations between the TLR ligands with/without Pru p 3 were carried out to study the synergistic effect between them. All of these experiments were done at 10 nM that it was shown to be effective in preliminary experiments. However, the immunological assays of the TLR_lig_ combinations (TLRn_lig_ with or without Pp3) assumed a double concentration (10 nM of TLR4_lig_ with or without Pp3 plus 10 nM TLR7_lig_ with or without Pp3). Moreover, LPS (Sigma-Aldrich) was included as positive controls (100 μg/mL) and complete medium as negative control, for 48 h at 37 °C in 5% CO_2_. After this, moDCs were harvested and their maturation status were assessed by analyzing the expression of CD80, CD83, CD86, and PD-L1 molecules (BD and Biolegend, San Diego, CA). Cells were acquired in a FACSCanto II Cytometer (BD), and data were analyzed using FlowJo software (Tree Star, Inc.). Results were expressed as maturation index (MI), as has been described.^69^

### Specific Proliferative Response of Different Cell Subpopulations

The specific proliferation of different lymphocyte subpopulations was evaluated using, as APCs, autologous moDCs pre-stimulated with 10 nM of different experimental combinations for 48 h previously described. Proliferation was determined using a 5,6-carboxyfluorescein diacetate *N*-succinimidyl ester (CFSE) dilution assay (Thermo Fisher Scientific). A total of 1.5 × 10^6^/mL pre-labeled CD14^–^ cells were cultured with moDCs pre-stimulated in different experimental combinations (10:1 ratio) at a final volume of 250 μL of complete medium in 96-well plates for 6 days at 37 °C and 5% CO_2_. 10 μg/mL phytohemaglutinin (Sigma-Aldrich) was used as positive proliferative control and unstimulated moDCs as negative proliferative control. The proliferative responses were assessed by flow cytometry, analyzing CFSElow expression in the different cell subsets as T-lymphocytes (CD3^+^T, CD4^+^T and CD8^+^T), B-lymphocytes (CD19^+^B) and NK-cells (CD56^+^NK). Results were expressed as PI. The PI was calculated for each cell subset as ([% CFSElow stimulated PBMCs + DC] – [% CFSElow stimulated PBMCs)/(% CFSElow unstimulated PBMCs + DCs)].^69,70^ In addition, the percentages of regulatory T (Treg) cells were also evaluated by flow cytometry, analyzing the expression of the CD4^+^CD127^–^/lowCD25^+^FOXP3^+^ markers.

### Cytokine Determination

To determine cytokine production from Pp3 or the different combination of TLR-Pp3-specific T-lymphocytes, the supernatants from moDCs (48 h of culture) and the lymphocyte cultures (6 days) were collected, and cytokine production (IL-4, IL-5, IL-10, and IFNγ) was determined with a human ProcartaPlex Multiplex Immunoassays kit (Thermo Fisher Scientific), following the manufacturer’s indications and detected in a Bio-Plex 200 (Bio-Rad, Hercules, CA). Data were analyzed using Bio-Plex Data Analysis Software (Bio-Rad, Hercules, CA).

### Statistic

The data were analyzed using the Shapiro–Wilk test to determine the normal distribution, but the most variables were fitted to non-parametric distribution. The Friedman test was used to find significant differences due to the effects of different TLRn_lig_-Pp3 between subjects from the same group. If the Friedman test indicated the existence of significant differences between treatments, we used the Wilcoxon signed rank test to compare between pairs of related samples, resulting in five post hoc tests (Pp3 vs TLRn_lig_-Pp3; Pp3 vs TLRn_lig_-Pp3 plus Pp3; TLRn_lig_-Pp3 vs TLRn_lig_; TLRn_lig_ vs TLRn_lig_ plus Pp3; and TLRn_lig_-Pp3 vs TLRn_lig_ plus Pp3). Bonferroni correction was used to reduce the threshold for significance to 0.01. We also compared the effects of the same treatment between the different groups of subjects (allergic patients vs tolerant controls) to examine the effect of Pp3 and the TLRn_lig_ with and without Pp3 using the Kruskal–Wallis test. This showed the existence of significant differences between groups; therefore, we applied Mann–Whitney *U* test to compare between allergic patients and tolerant controls groups receiving the same treatment. This showed four post hoc tests reducing the threshold for significance to 0.0125, Bonferroni correction.

In addition, to examine the effect of the combination both TLRn_lig_-Pp3 between allergic patients versus tolerant control, the statistical analysis was the same, the Kruskal–Wallis test followed by the Mann–Whitney *U* test, to show the existence of significant differences between groups. This resulted in three post hoc tests. Bonferroni correction was used to reduce the threshold for significance to 0.016. In addition, the Friedman test was used to find significant differences due to the effect of the combination both TLRn_lig_-Pp3 between subjects from the same group. In the case that the Friedman test indicated the existence of significant differences between treatments, we used the Wilcoxon signed rank test to compare between pairs of related samples, resulting in three post hoc tests. Bonferroni correction was used to reduce the threshold for significance to 0.016.

## Supporting Information Available

Synthetic procedures and characterization of all new compounds, additional proliferation of cell population figures, and the clinical characteristics of allergy and control samples (PDF)

## Abbreviations

AIT: allergen-specific immunotherapy
CM: confocal microscopy
CTLs: C-type lectins
CuAAC: Cu(I) catalyzed azide–alkyne 1,3 dipolar cycloaddition
DCs: dendritic cells
DIPEA: diisopropylethylamine
DMF: dimethylformamide
DMEN: *N*,*N*-dimethylethylenediamine
DMSO: dimethylsulfoxide
FA: food allergy
HATU: 1-[bis(dimethylamino)methylene]-1*H*-1,2,3-triazolo[4,5-*b*]pyridinium 3-oxid hexafluorophosphate
IL-10: interleukine 10
LTP: lipid transfer protein
MPLA: monophosphoryl-lipid A
moDCs: monocyte-derived DCs
OVA: ovalbumin
PAMPs: pathogen associated molecular patterns
PEG: polyethylene glycol
PPRs: pattern-recognition receptors
TLR: toll-like receptor
