# Supplementary material for: Immunomodulatory Response of Toll-like Receptor Ligand–Peptide Conjugates in Food Allergy
Source: ACS Chem Biol. 2021 Nov 11;16(11):2651–64. doi: 10.1021/acschembio.1c00765 (PMC8609526; doi:10.1021/acschembio.1c00765)
Supplement: Supplementary file 1 — cb1c00765_si_001.pdf [file cb1c00765_si_001.pdf]

Supporting Information

The immunomodulatory response of Toll-like receptor ligand-peptide conjugates in food allergy

Jorge Losada Méndez[a], Francisca Palomares[b], Francisca Gómez[c], Pedro Ramírez-López[a,e], Javier Ramos-Soriano[a], Maria Jose Torres,[c] Cristobalina Mayorga,[b-d,]* and Javier Rojo[a,]*

[a]*Glycosystems Laboratory, Instituto de Investigaciones Químicas (IIQ), CSIC – Universidad de Sevilla, 41092 Seville, Spain.*

[b]*Allergy Unit, IBIMA, Regional University Hospital of Malaga, UMA, 29009 Malaga, Spain.*

[c] *Allergy Clinical Unit, Hospital Regional Universitario de Málaga, 29009 Málaga, Spain.*

[d] *Nanostructures for Diagnosing and Treatment of Allergic Diseases Laboratory, Centro Andaluz de Nanomedicina y Biotecnología-BIONAND, 29590 Málaga, Spain.*

[e)] *Present address: Chemistry in Pharmaceutical Sciences Department. Complutense University of Madrid, Plaza Ramón y Cajal s/n, 28040 Madrid, Spain.*

Email addresses: mayorga.lina@gmail.com and javier.rojo@iiq.csic.es

1. Materials for Synthesis
2. Synthetic procedures
3. Figure S1
4. Figure S2
5. Table S1

1. Materials for Synthesis

Chemicals were purchased from Sigma-Aldrich, Merck, Acros Organics and used without further purification. Pp3 peptide was synthesized at Peptide Synthesis Facility, Department of Experimental and Health Science, Pompeu Fabra University, Barcelona, Spain. Solvents were purchased from Fisher scientific, ScharLab and Carlo Erba. Anhydrous solvents were purchased from Sigma-Aldrich®. H_2O was purified with a Milli-Q purification system from Millipore (18.3 Ω). When dry solvents were employed, reactions were performed under Ar atmosphere. TLC were performed using pre-coated aluminum chromate-plates Silica Gel 60 F254 Merck of 0.25 mm thick. Purifications by column chromatography were performed using Silica gel 60 (particle size 0,063-0,200 nm or 0,015-0,040 mm), from Merck, eluting by gravity or subjecting it to light pressure. NMR spectra, ^{1}H and ^{13}C, were recorded at 298 K on a Bruker DRX400 spectrometer. HSQC NMR experiments were carried out to assist in signal assignment. All chemical shifts were reported in ppm (δ) using the residual proton solvent peaks as internal standards. These abbreviations were used to indicate the

multiplicities: s = singlet, d = doublet, t = triplet, m = multplet, brs =m broad singlet. ESI Mass spectra were obtained with an Esquire 6000 ESI-Ion Trap (Bruker Daltonics). For ultrafiltration, Amicon Ultra-15, MWCO 3KDa centrifugal filters from Merck were employed. Analytical HPLC was carried out on column BioZen, 2.6 µm, Peptide XB-C18 or BioZen, 2.6 µm, Peptide XB-C18 with a Waters Aliance 2690. Solvents A and B were 0.05% and 0.1% (*v*/*v*) TFA in H_2O and CH3CN, respectively. Elution was performed with linear gradients of solvent B into A over 15 min, at a 1 mL min^{-1} flow rate.

2. Synthetic Procedures

Compound **3** was synthesized according to Zysman-Colman E. *et al.*[1] Compounds **5-7** were synthesized according to Chan M. *et al.*[2] and compound **15** was synthesized according to Bakleh M. E. *et al.*[3]

Ethyl 2-((4-Bromo-2-cyanophenyl)amino)acetate (4)

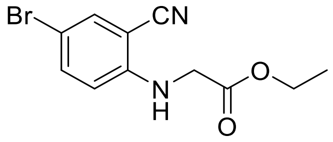

The aniline derivative **3** (8.18g, 41.51 mmol), ethyl bromoacetate (41.59 g, 249 mmol) and potassium carbonate (6.31 g, 45.66 mmol) were combined in anhydrous DMF at 60°C for 2 days. Then, the solvent was removed under high vacuum and the crude was purifed by column chromatography on silica gel (Hexane/ ethyl acetate, 8:1) affording **4** (7.37g, 63%) as a white solid. ^{1}H NMR ($CDCl_3$, 400 MHz) δ: 7.53 (ArC<u>H</u>, d, *J* = 2.3 Hz, 1H), 7.49 (dd, *J* = 8.9, 2.3 Hz, 1H, ArC<u>H</u>), 6.47 (d, *J* = 8.9 Hz, 1H, ArC<u>H</u>), 5.23 (brs, 1H, N<u>H</u>), 4.28 (q, *J* = 7.1 Hz, 2H, C$\underline{H_2}$$CH_3$), 3.98 (d, *J* = 5.5 Hz, 2H, NHC$\underline{H_2}$), 1.32 (t, *J* = 7.1 Hz, 3H, CH_2C$\underline{H_3}$). ^{13}C-NMR (100 MHz, CDCl3) δ: 169.4, 148.2, 137.2, 134.8, 116.1, 112.5, 108.4, 98.2, 61.9, 45.0, 14.2.

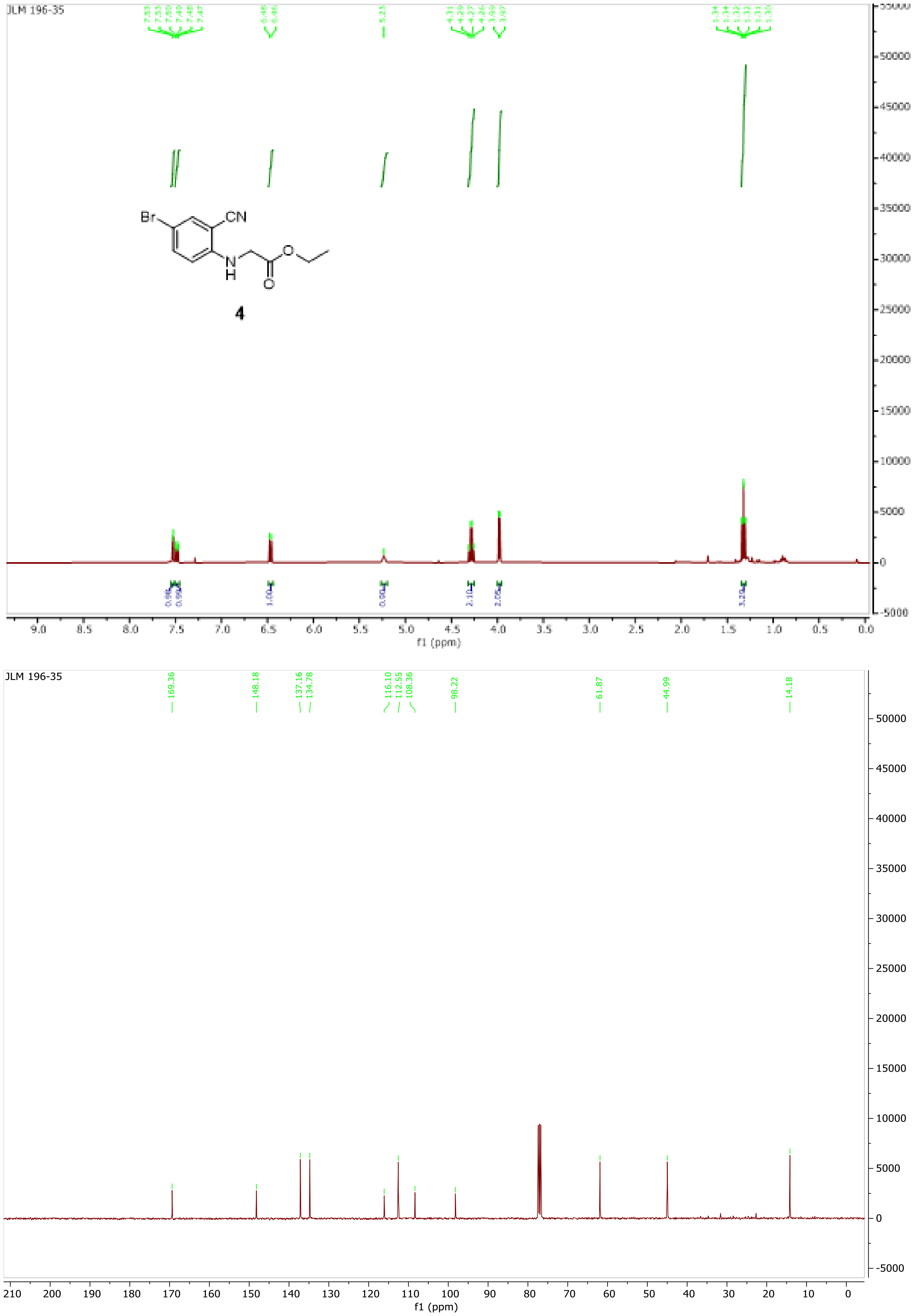

^{1}H-RMN (400 MHz, $CDCl_3$, 298 K) spectrum (up) and ^{13}C-RMN (100 MHz, $CDCl_3$, 298 K) spectrum (down) of compound **4**.

2-((8-bromo-4-oxo-3-phenyl-4,5-dihydro-3H-pyrimido[5,4-b]indol-2-yl)thio)acetic acid (8)

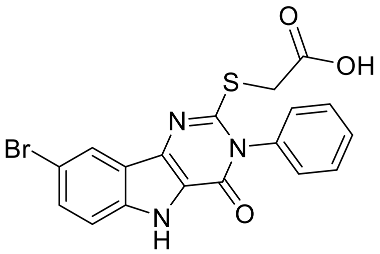

In a schlenk flask, to a solution of **7** (697 mg, 1.87 mmol) in warm EtOH (15 mL) was added potassium hydroxide (210 mg, 3.74 mmol) with stirring. To this solution was added a solution of bromoacetic acid (269 mg, 1.87 mmol mmol) in 3 mL of EtOH and the mixture was refluxed overnight. Then, about half volume of the mixture was removed on a rotavap and the mixture was acidified to pH= 4 with 4M HCl. The solid was filtered and washed with distilled water and finally with EtOH, to give compound **8** as a white solid (810 mg, quant.). ^{1}H NMR (DMSO-d$_6$, 400 MHz) δ: 12.36 (s, 1H, N<u>H</u>), 8.09 (s, 1H, ArC<u>H</u>), 7.61-7.49 (m, 7H, ArC<u>H</u>), 3.96 (s, 2H, SC<u>H</u>$_2$). ^{13}C-NMR (100 MHz, DMSO-d$_6$) δ: 170.3, 155.3, 153.5, 137.9, 136.6, 136.3, 130.5, 130.3, 130.1, 129.9, 122.7, 122.4, 120.7, 115.6, 112.9, 35.7. ESI-MS m/z calcd. for $C_{18}H_{12}O_3N_3BrS$: 430.3 [M]$^+$; found: 432.1. [M + H]$^+$. ESI-HRMS m/z calcd. for $C_{18}H_{11}O_3N_3BrNaS$: 451.9680; found: 451.9675.

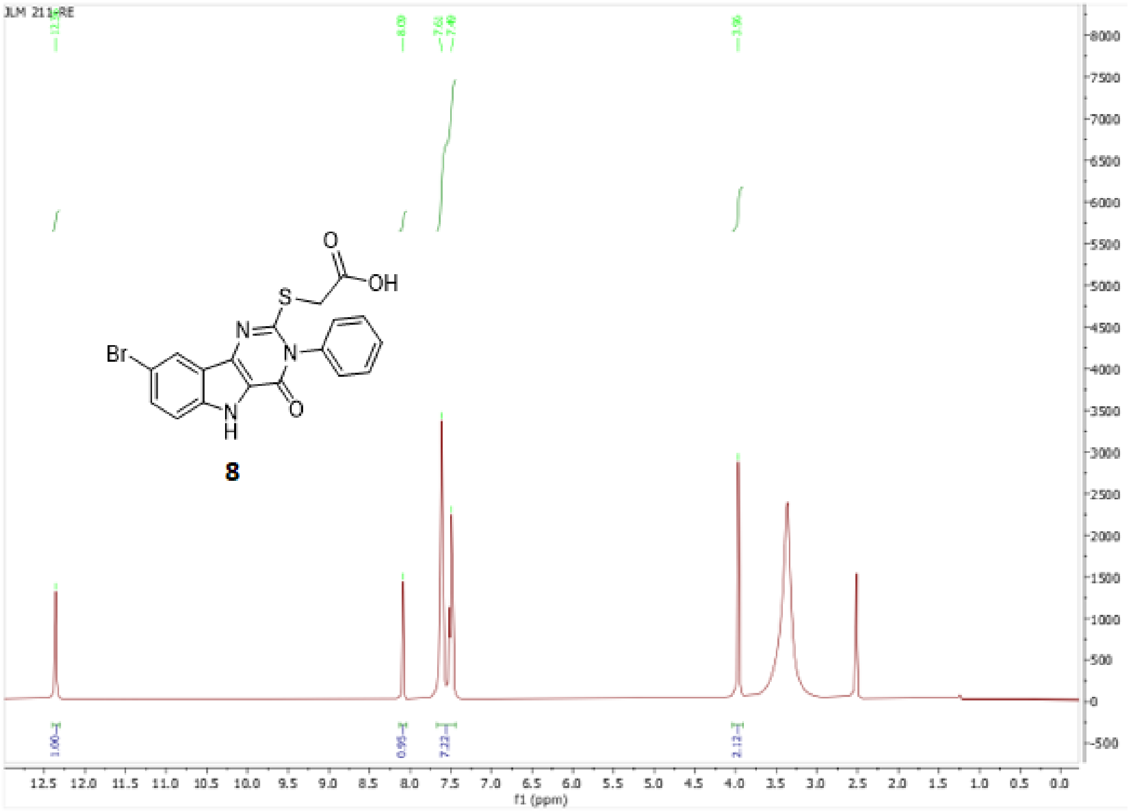

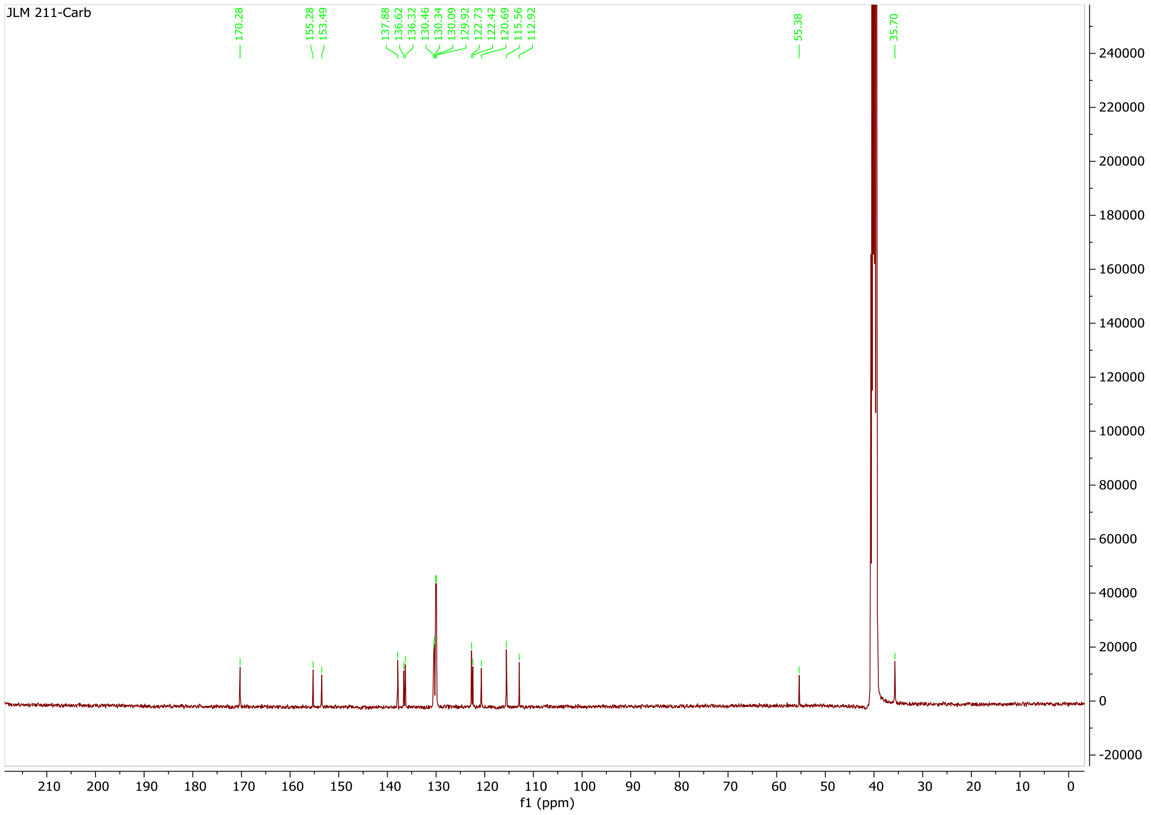

^{1}H-RMN (400 MHz, DMSO-d$_6$, 298 K) spectrum (up) and ^{13}C-RMN (100 MHz, DMSO-d$_6$, 298 K) spectrum (down) of compound **8**.

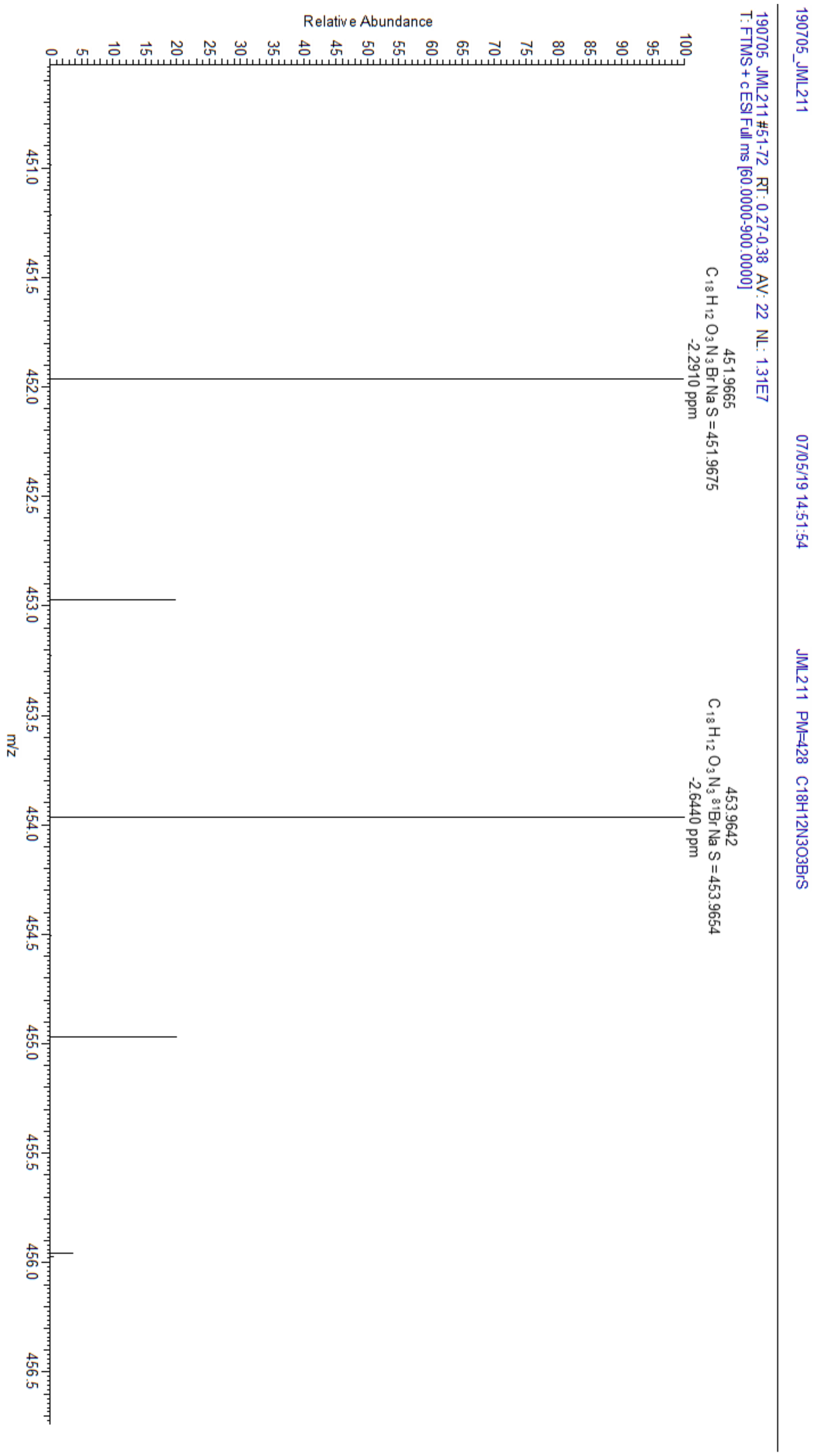

ESI-HRMS spectrum of compound **8**.

2-((8-bromo-4-oxo-3-phenyl-4,5-dihydro-3H-pyrimido[5,4-b]indol-2-yl)thio)-*N*-cyclohexylacetamide (9)

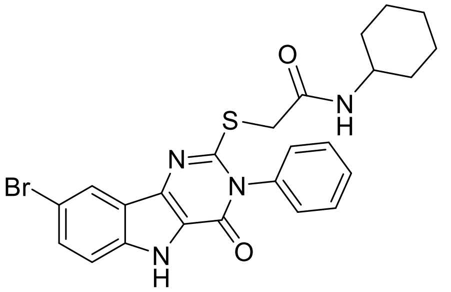

Compound **8** (1.732 g, 4.025 mmol), anhydrous triethylamine (1.12 mL, 8.05 mmol), and cyclohexylamine (0.51 mL, 4.428 mmol) were dissolved in anhydrous DMF (29 mL). To this solution, HATU (823 mg, 2.16 mmol) dissolved in anhydrous DMF (5.7 mL) was added and stirred at room temperature for 6 h. The solvent was removed under high vacuum and the crude material was washed twice with MeOH yielding compound **9** as a white solid (2.036 g, quant). ^{1}H NMR (DMSO-d$_6$, 400 MHz) δ: 12.32 (s, 1H, NH), 8.29 (s, 1H, OCNH), 8.22 (d, *J* = 7.6 Hz, 1H, ArCH), 7.61-7.49 (m, 7H, ArCH), 3.86 (s, 2H, SCH$_2$), 3.54 (m, 1H, overlap water signal, HNCH), 1.75-1.54 (m, 5H, cyclohexyl), 1.25 (m, 5H, cyclohexyl). ^{13}C-NMR (100 MHz, DMSO-d$_6$) δ: 166.3, 155.3, 153.8, 137.9, 136.8, 136.4, 130.4, 130.2, 130.0, 129.9, 123.3, 122.5, 120.7, 115.4, 112.8, 48.5, 37.2, 33.0, 25.7, 25.0. ESI-MS m/z calcd. for $C_{24}H_{23}O_2N_4BrS$: 510.1 [M]$^+$; found: 511.1 [M + H]$^+$. ESI-HRMS m/z calcd. for $C_{24}H_{23}O_2N_4BrNaS$: 533.0623; found: 523.0617.

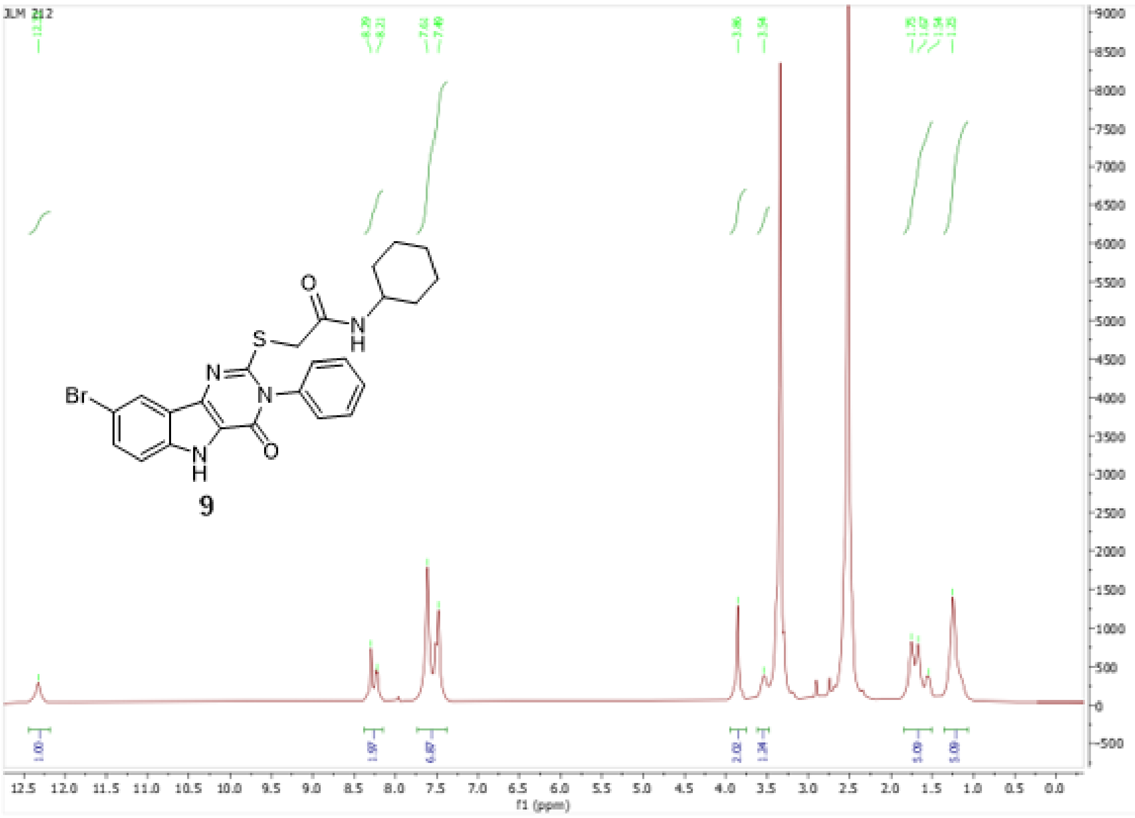

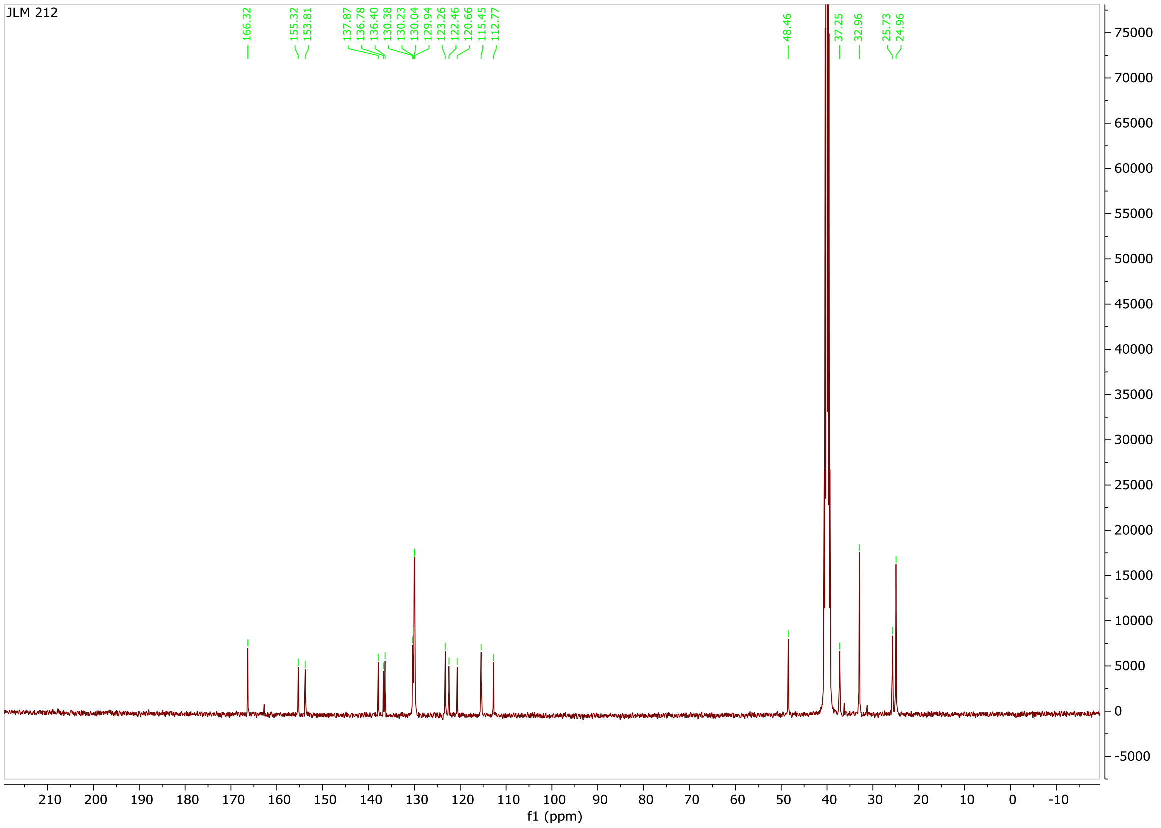

^{1}H-RMN (400 MHz, DMSO-d_6, 298 K) spectrum (up) and ^{13}C-RMN (100 MHz, DMSO-d_6, 298 K) spectrum (down) of compound **9**.

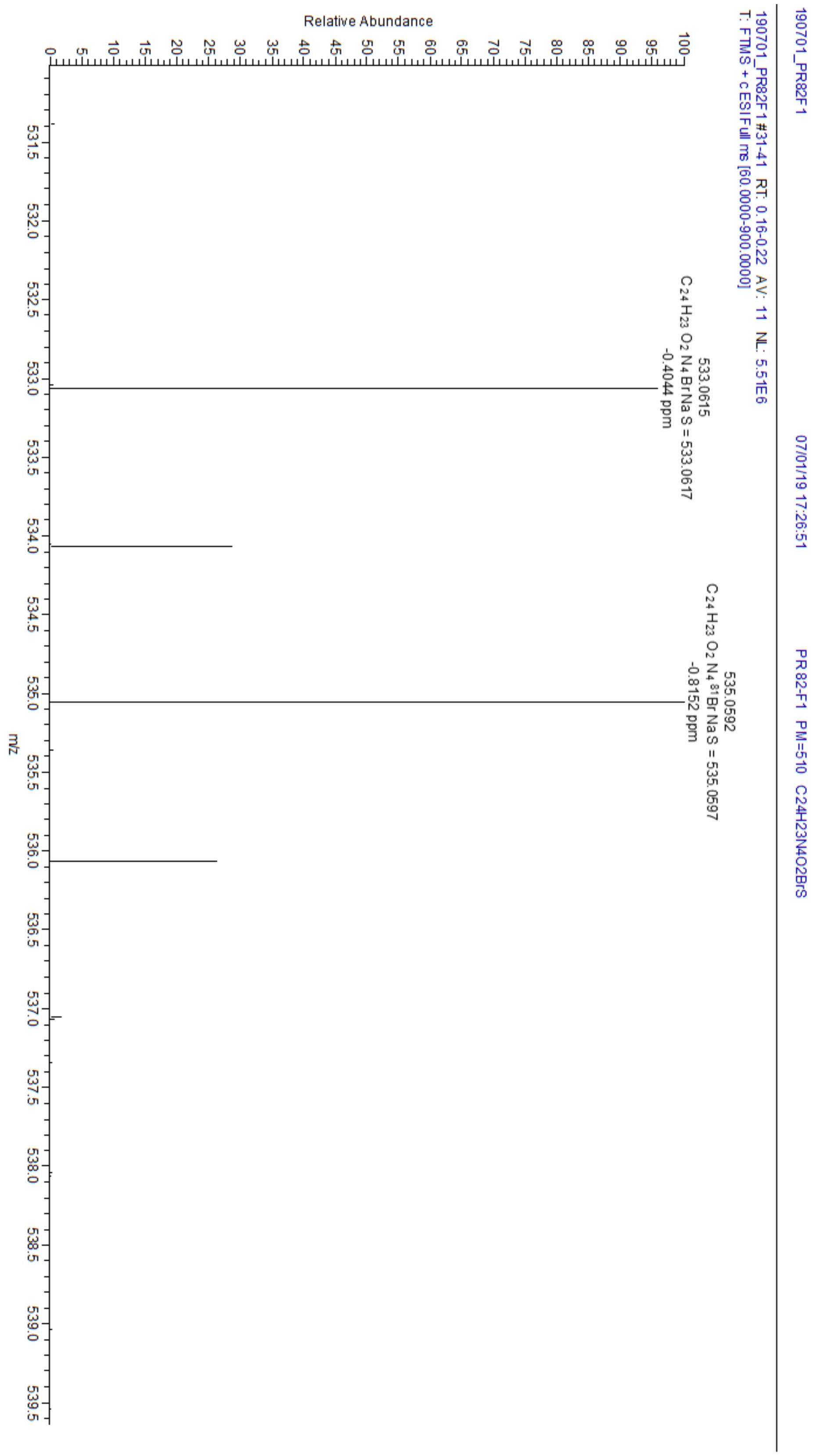

ESI-HRMS spectrum of compound **9**.

2-((8-amino-4-oxo-3-phenyl-4,5-dihydro-3H-pyrimido[5,4-b]indol-2-yl)thio)-*N*-cyclohexylacetamide (10)

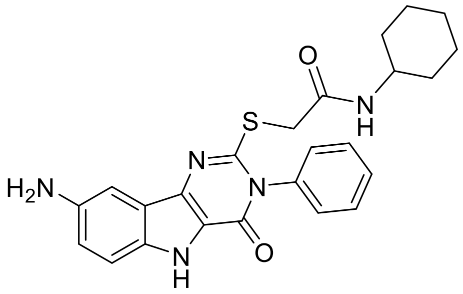

Compound **9** (295 mg, 0.58 mmol), sodium azide (375 mg, 5.77 mmol), CuI (549 mg, 2.88 mmol), and sodium ascorbate (286 mg, 1.44 mmol) were combined in a flask under argon atmosphere. A mixture of DMSO:H_2O (5:1, 9.1 mL) was degassed with argon and added to the mixture, followed by the addition of *N*,*N*-dimethylethylenediamine (310 μL, 2.88 mmol) degassed too. The reaction was heated to 90 °C overnight with stirring under argon atmosphere. The suspension was extracted with EtOAc and brine. The organic layer was dried over anhydrous $MgSO_4$ and concentrated under vacuum. The crude residue was purified by column chromatography on silica gel (DCM:MeOH, 100/3) affording **10** as a brown pale solid (185 mg, 72%). ^{1}H NMR (DMSO-d_6, 400 MHz) δ: 11.61 (s, 1H, N<u>H</u>), 8.12 (d, *J* = 7.3 Hz, 1H, OCN<u>H</u>), 7.59 (m, 3H, ArC<u>H</u>), 7.44 (m, 2H, ArC<u>H</u>), 7.24 (m, 1H, ArC<u>H</u>), 7.13 (s, 1H, ArC<u>H</u>), 6.90 (d, *J* = 8.5 Hz, 1H, ArC<u>H</u>), 4.90 (brs, 2H, N<u>H</u>$_2$), 3.89 (s, 2H, SC<u>H</u>$_2$), 3.50 (m, 1H, overlap water signal, HNC<u>H</u>), 1.74-1.66 (m, 4H, cyclohexyl), 1.52 (m, 1H, cyclohexyl), 1.25-1.11 (m, 5H, cyclohexyl). ^{13}C-NMR (100 MHz, DMSO-d_6) δ: 166.1, 155.4, 151.2, 142.9, 136.7, 133.3, 130.3, 130.2, 130.1, 129.9, 121.7, 119.6, 119.1, 113.5, 102.0, 48.4, 37.2, 32.7, 25.6, 24.9. ESI-S m/z calcd. for $C_{24}H_{25}O_2N_5S$: 447.2 $[M]^+$; found: 448.1 $[M + H]^+$, 470.1 $[M + Na]^+$, 917.2 $[2M + Na]^+$. ESI-HRMS m/z calcd. for $C_{24}H_{26}O_2N_5S$: 448.1807; found: 448.1802.

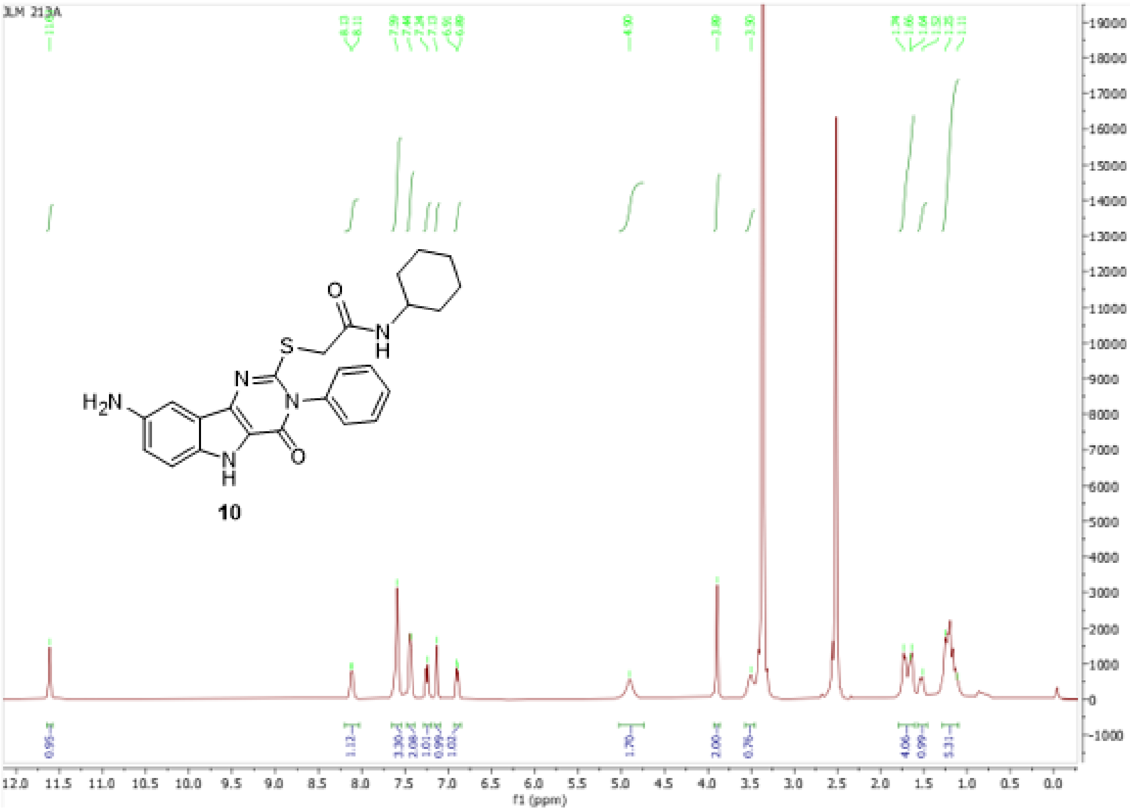

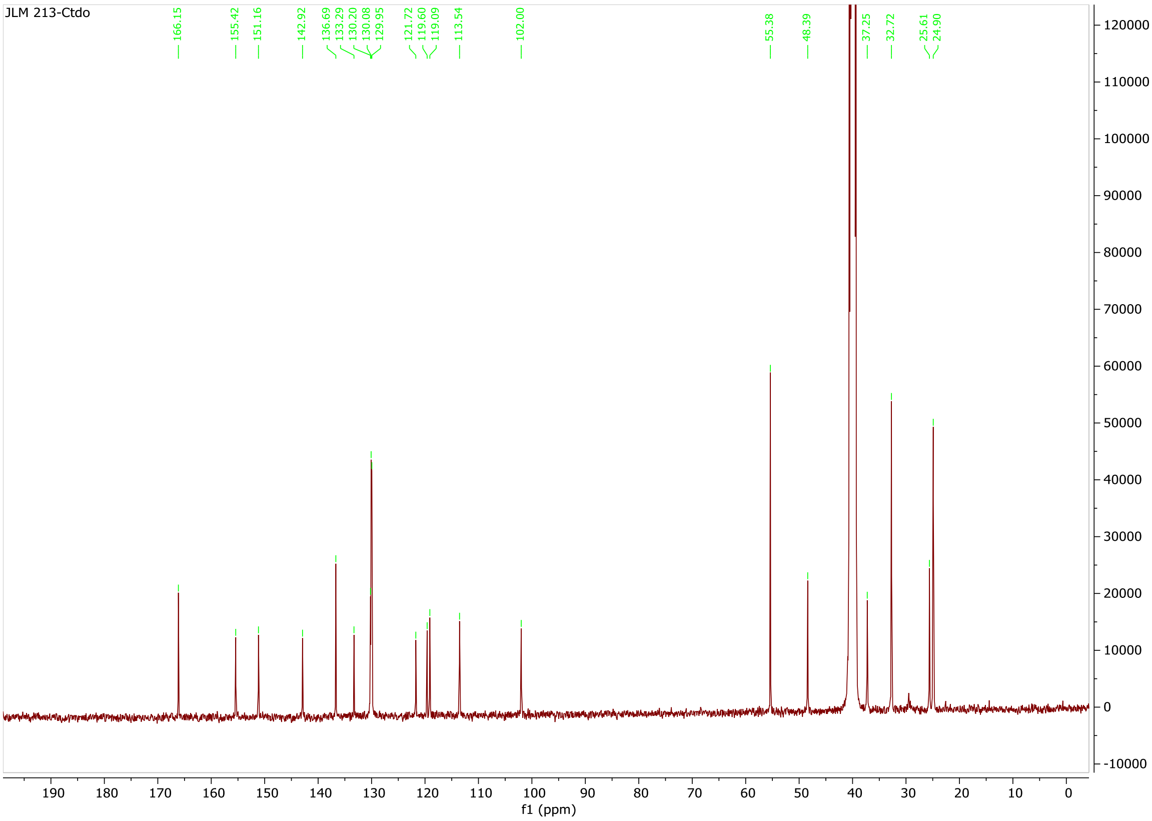

^{1}H-RMN (400 MHz, DMSO-d_6, 298 K) spectrum (up) and ^{13}C-RMN (100 MHz, DMSO-d_6, 298 K) spectrum (down) of compound **10**.

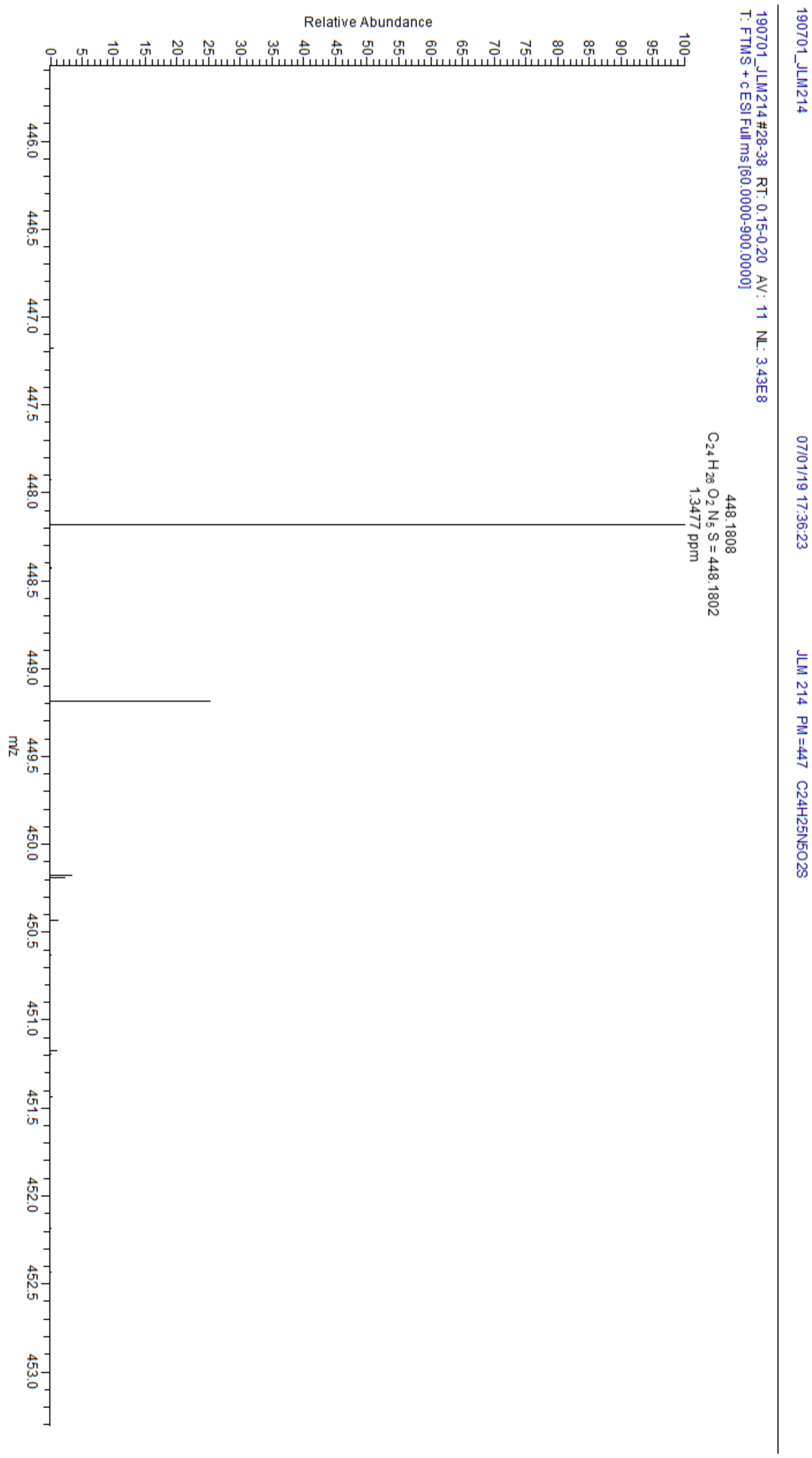

ESI-HRMS spectrum of compound **10**.

***N*-(2-((2-(cyclohexylamino)-2-oxoethyl)thio)-4-oxo-3-phenyl-4,5-dihydro-3H-pyrimido[5,4-b]indol-8-yl)-3-(2,5-dioxo-2,5-dihydro-1H-pyrrol-1-yl)propanamide (11)**

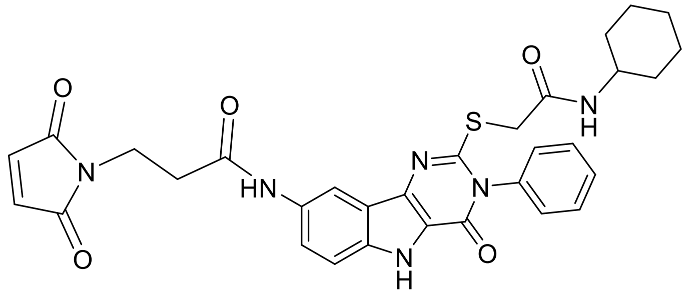

Compound **10** (185 mg, 0.41 mmol), anhydrous DIPEA (144 µL, 0.83 mmol), 3-maleimidopropionic acid,[19] (77 mg, 0.45 mmol) were dissolved in anhydrous DMF (3 mL). To this solution, HATU (173 mg, 0.45 mmol) dissolved in anhydrous DMF (1.7 mL) was added and stirred at room temperature overnight. The solvent was removed under high vacuum and the crude material was washed twice with MeOH yielding **11** as a white solid (182 mg, 73%). ^{1}H NMR (DMSO-d$_6$, 400 MHz) δ: 12.05 (s, 1H, N<u>H</u>), 10.06 (s, 1H, OCN<u>H</u>-Ar), 8.49 (s, 1H, ArC<u>H</u>), 8.21 (brs, 1H, OCN<u>H</u>), 7.60-7.42 (m, 7H, ArC<u>H</u>), 7.05 (s, 2H, (OCC<u>H</u>)$_2$N), 3.94 (s, 2H, SC<u>H$_2$</u>), 3.77 (t, *J* = 7.2 Hz, 2H, NC<u>H$_2$</u>CH$_2$), 3.50 (m, 1H, overlap water signal, HNC<u>H</u>), 2.64 (t, *J* = 7.2 Hz, 2H, NCH$_2$C<u>H$_2$</u>), 1.74-1.54 (m, 5H, cyclohexyl), 1.21-1.10 (m, 5H, cyclohexyl). ^{13}C-NMR (100 MHz, DMSO-d$_6$) δ: 171.3, 168.6, 165.9, 155.4, 152.7, 137.6, 136.6, 136.1, 135.1, 132.54, 130.3, 130.1, 130.0, 121.7, 120.6, 120.4, 113.4, 110.8, 48.5, 37.5, 35.4, 34.4, 32.7, 25.6, 24.9. ESI-MS m/z calcd. For $C_{31}H_{30}O_5N_6S$: 598.2 [M]$^+$; found: 621.2 [M + Na]$^+$. ESI-HRMS m/z calcd. for $C_{31}H_{30}O_5N_6NaS$: 621.1896; found: 621.1891. RP-HPLC t_R = 14.31 min (Column: analytical BioZen, 2.6 µm, Peptide XB-C18; A: H_2O (0.05 % TFA), B: Acetonitrile (0.1 % TFA); first two minutes with 10% of B, the rest, 10 to 95% linear gradient of B into A over 15 min, F = 1 mL/min).

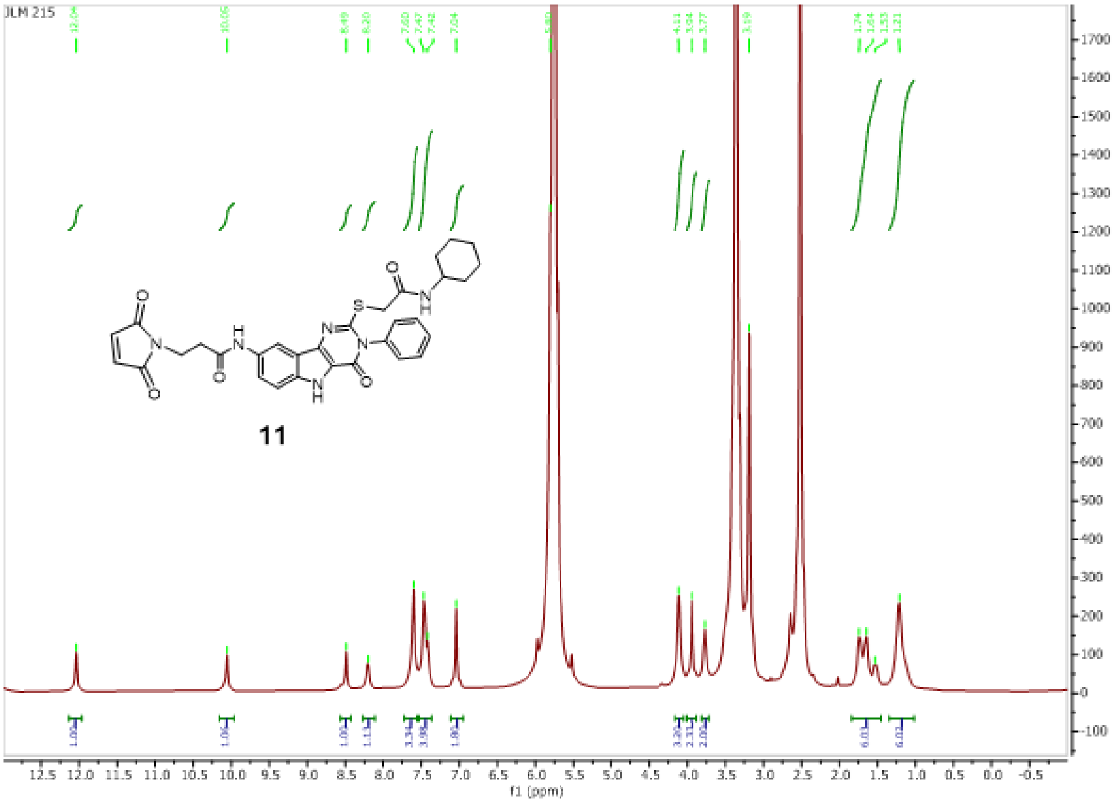

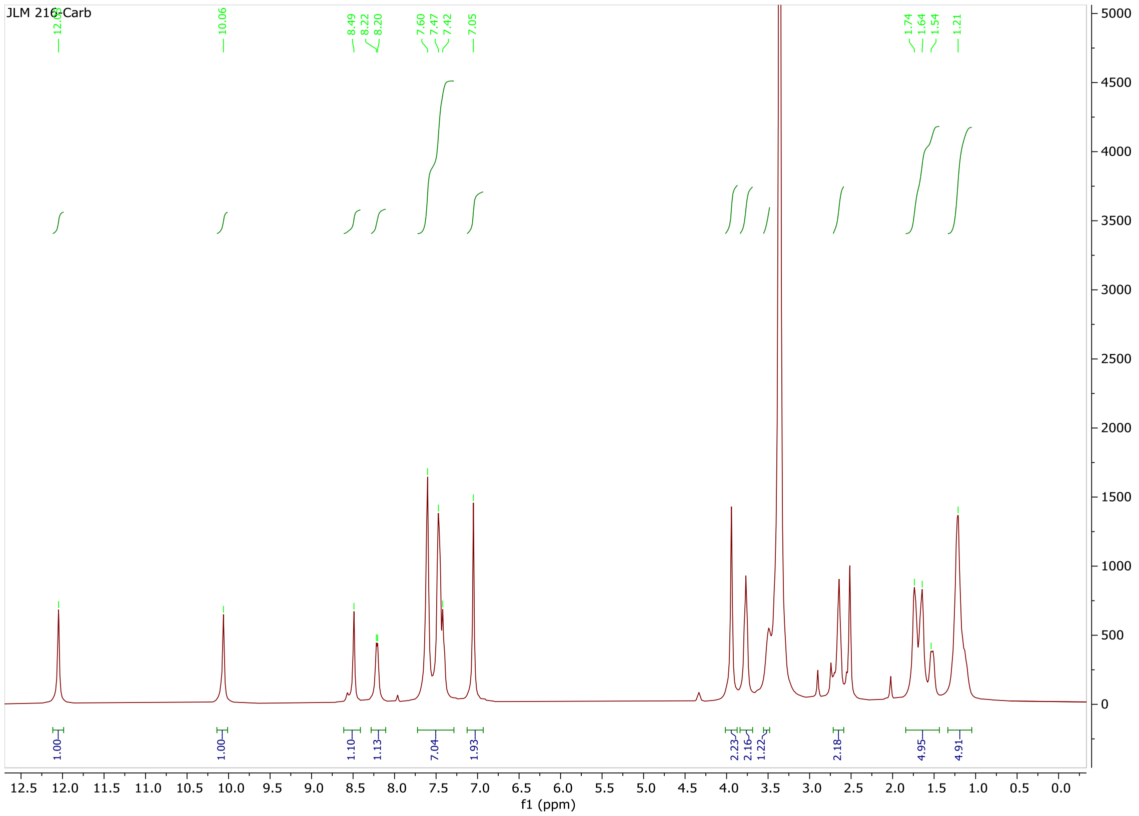

^{1}H-RMN (400 MHz, DMSO-d_6, 298 K) spectrum (up) and ^{13}C-RMN (100 MHz, DMSO-d_6, 298 K) spectrum (down) of compound **11**.

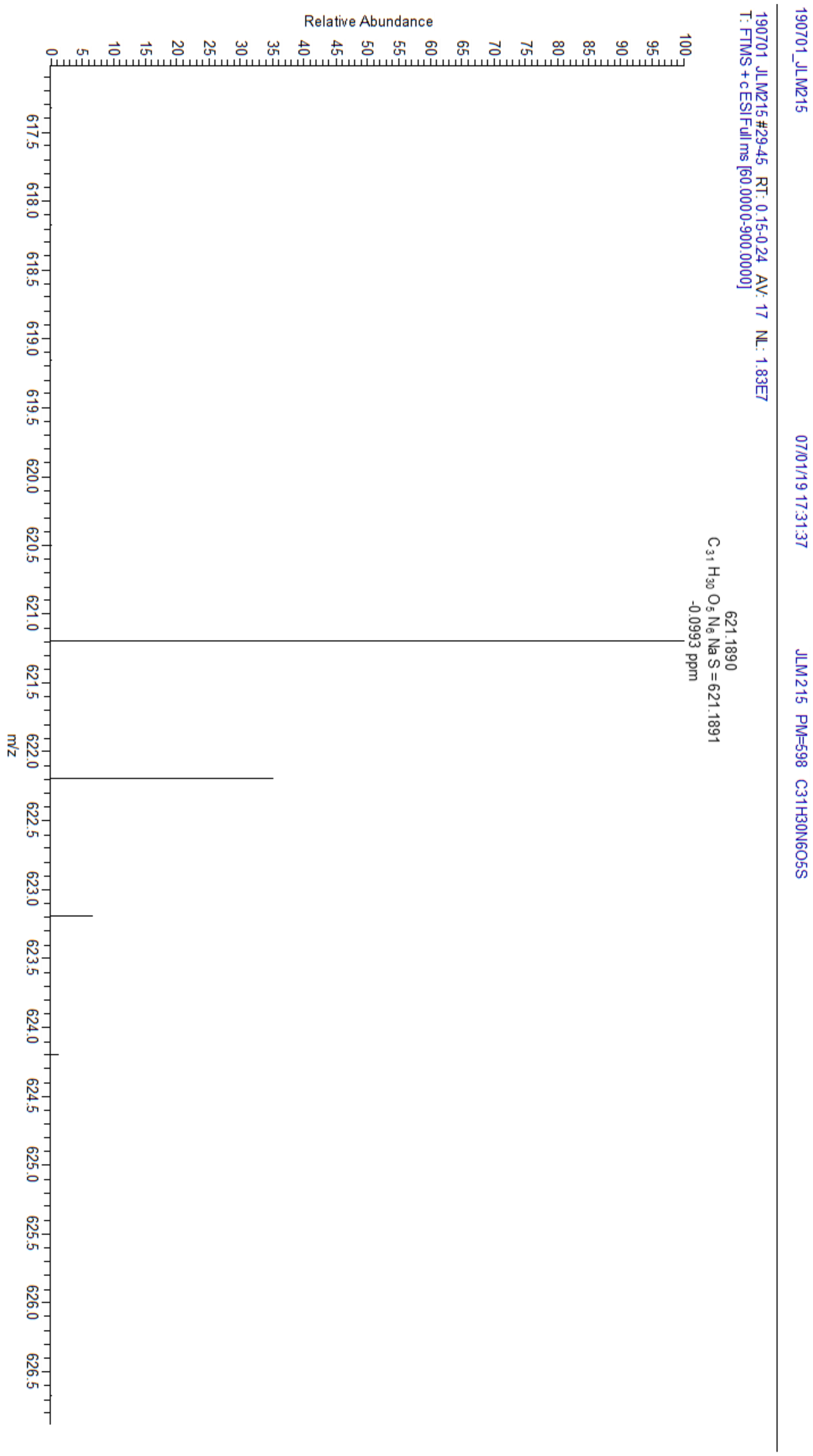

ESI-HRMS spectrum of compound **11**.

TLR4$_{lig}$-Pp3 (1)

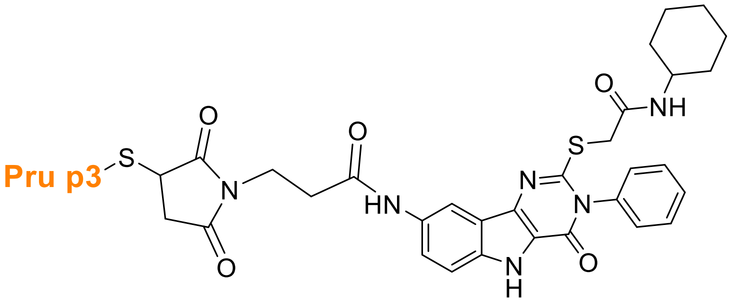

Following the general procedure, using maleimide derivative **11** as starting material, conjugate **1** was obtained as a white solid (2.79 mg, 85%). ESI-MS m/z calcd.: 2853.8 $[M]^+$; found: 952.5 $[M + 3H]^{+3}$, 1427.8 $[M + 2H]^{+2}$. RP-HPLC: t_R = 10.65 min (Column: analytical BioZen, 2.6 µm, Peptide XB-C18; A: H_2O (0.05 % TFA), B: Acetonitrile (0.1 % TFA); first two minutes with 10% of B, the rest, 10 to 95% linear gradient of B into A over 15 min, F = 1 mL/min).

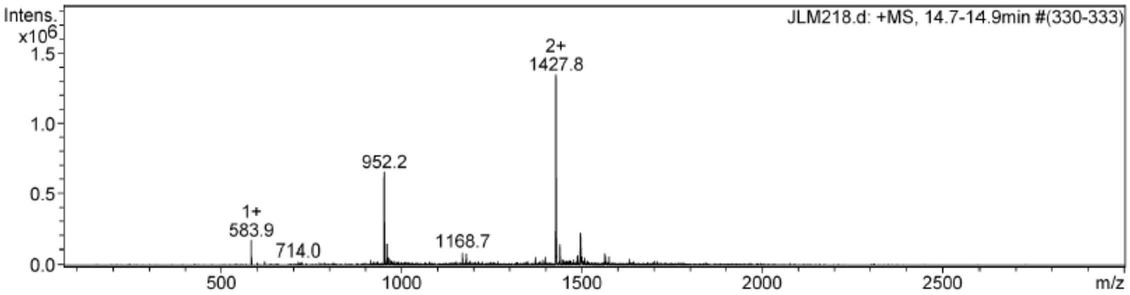

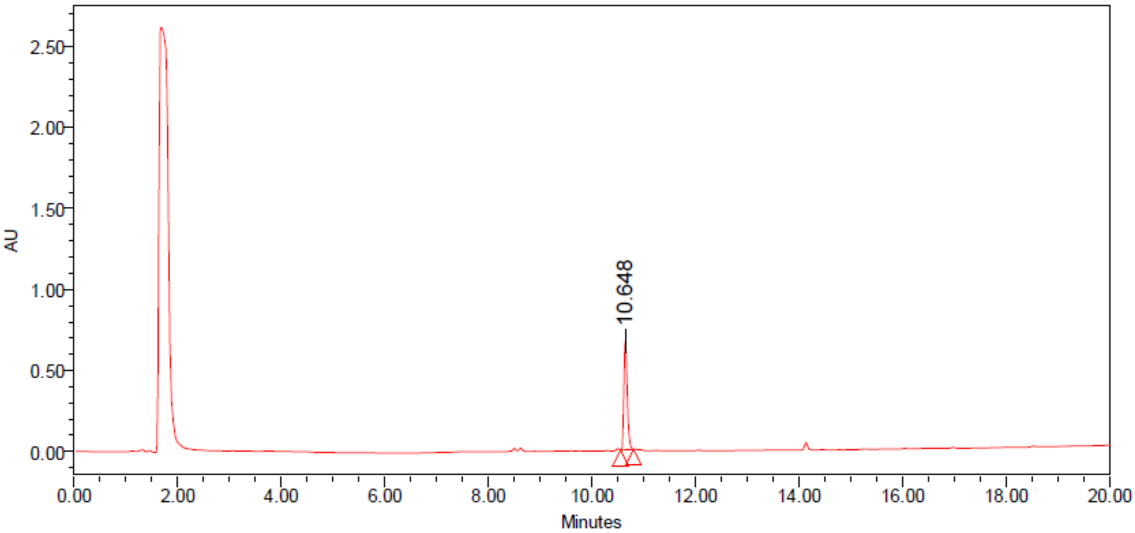

_N_-(2-(2-(2-(2-azidoethoxy)ethoxy)ethoxy)ethyl)-4-(bromomethyl)benzamide (16)

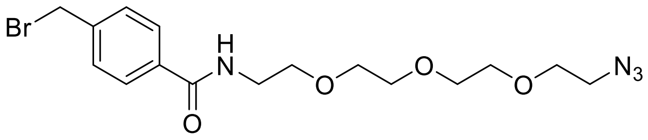

4-Bromometilbenzoic acid (1.5 g, 6.9 mmol) was reacted with thionyl chloride (15.18 ml, 209.25 mmol). The reaction was refluxed for 3 hours and then, concentrated under reduced pressure.To the crude redissolved in 20 ml of anhydrous DCM at 0ºC was added dropwise a solution of the linker amino azide **15** (2.28g, 10.46 mmol) in anhydrous DCM (10 mL). The reaction was stirred overnight at r.t and the mixture was washed with 1M HCl, saturated solution $NaHCO_3$ and H_2O, and dried over anhydrous magnesium sulfate. The organic layer was concentrated under reduced pressure and purified by column chromatography on silica gel (Hexane/ ethyl acetate, 1:1) to afford **16** as a yellow oil (1.72 g, 60%). The product was obtained as an approx. 1:1 mixture of Cl/Br derivatives. ^{1}H NMR ($CDCl_3$, 400 MHz) δ: 7.80 (d, J = 8.2 Hz, 2H, ArC<u>H</u>), 7.48 (d, J = 8.2 Hz, 2H, ArC<u>H</u>), 6.79 (brs, 1H, N<u>H</u>), 4.53/4.63 (s, 2H, C<u>H</u>$_2$Br/Cl), 3.59-3.72 (m, 14H, C<u>H</u>$_2$PEG), 3.36 (t, J = 5.0 Hz, 2H, C<u>H</u>$_2$N$_3$). ^{13}C-NMR (100 MHz, $CDCl_3$) δ: 166.8, 141.1, 134.6, 129.1, 128.6, 127.6, 127.5, 70.7, 70.2, 70.0, 69.8, 50.6, 45.6, 39.8, 32.4. ESI-MS m/z calcd. For $C_{16}H_{23}O_4N_5Br$: 414.1 $[M]^+$; found: 437.1 $[M + Na]^+$. ESI-MS m/z calcd. For $C_{16}H_{23}O_4N_5Cl$: 370.1 $[M]^+$; found: 393.2 $[M + Na]^+$.ESI-HRMS m/z calcd. for $C_{16}H_{23}O_4N_5BrNa$: 437.0803; found: 437.0795. ESI-HRMS m/z calcd. for $C_{16}H_{23}O_4N_5ClNa$: 393.1308; found: 393.1300.

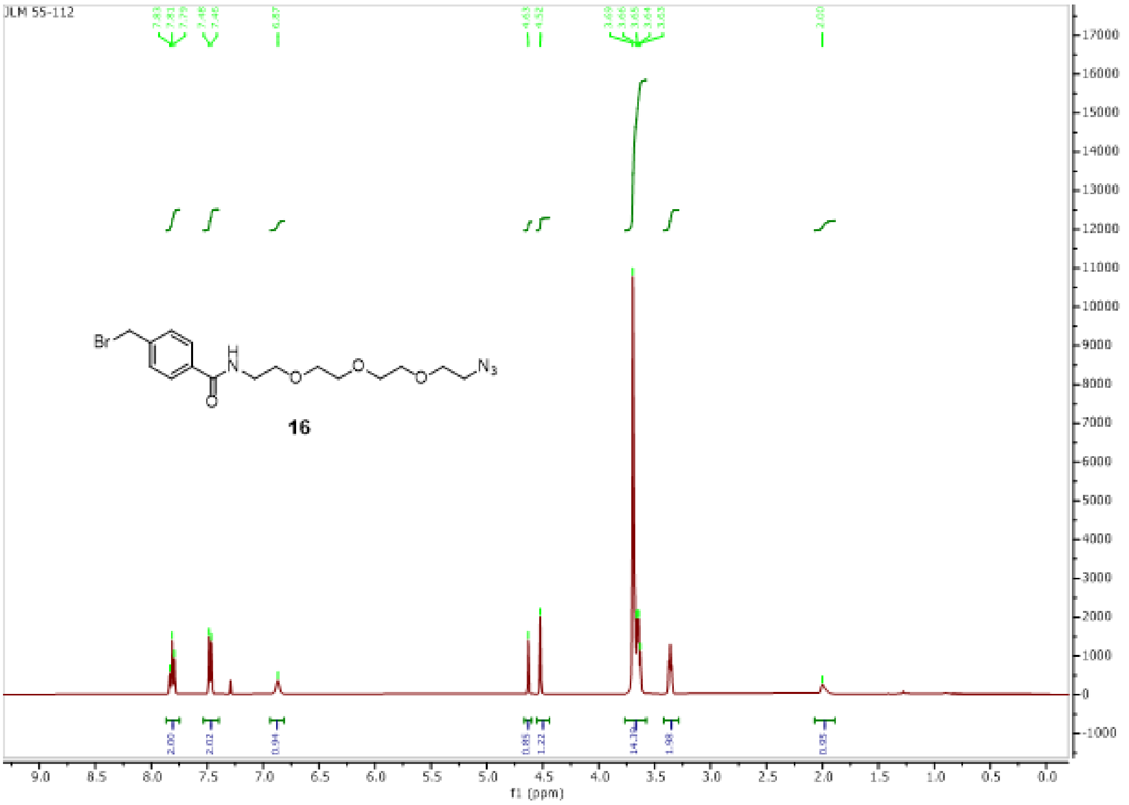

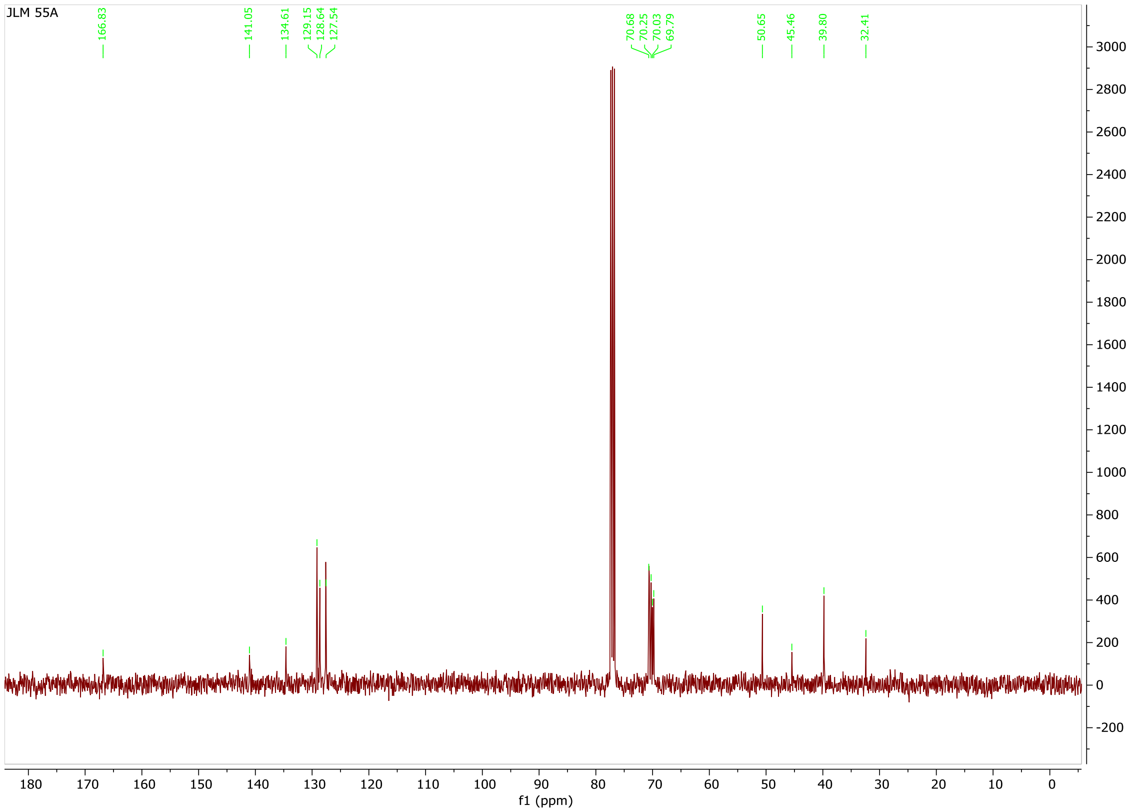

^{1}H-RMN (400 MHz, $CDCl_3$, 298 K) spectrum (up) and ^{13}C-RMN (100 MHz, $CDCl_3$, 298 K) spectrum (down) of compound **16**.

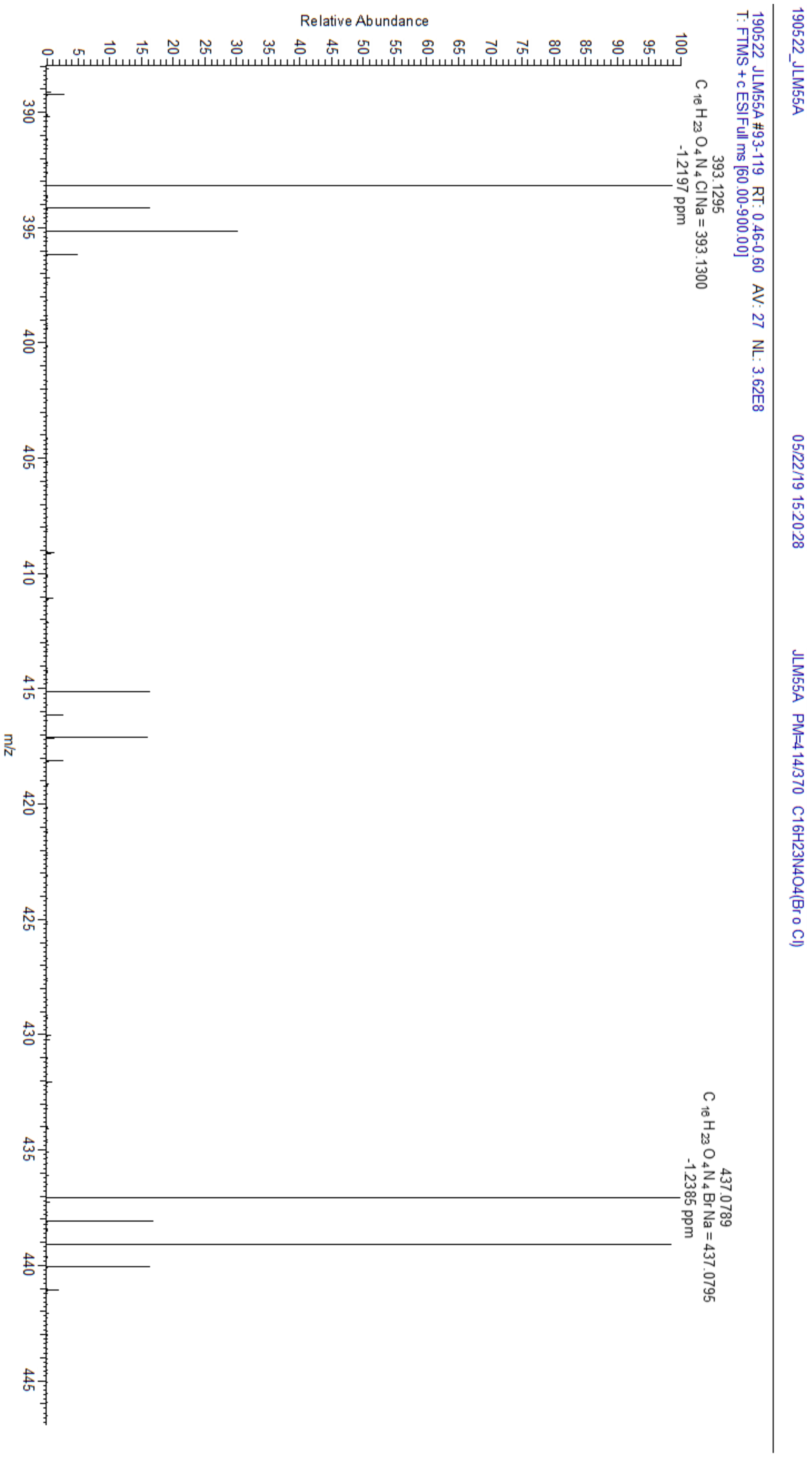

ESI-HRMS spectrum of compound **16**.

4-((6-amino-2-butoxy-9H-purin-9-yl)methyl)-N-(2-(2-(2-(2-azidoethoxy)ethoxy) ethoxy)ethyl)benzamide (18)

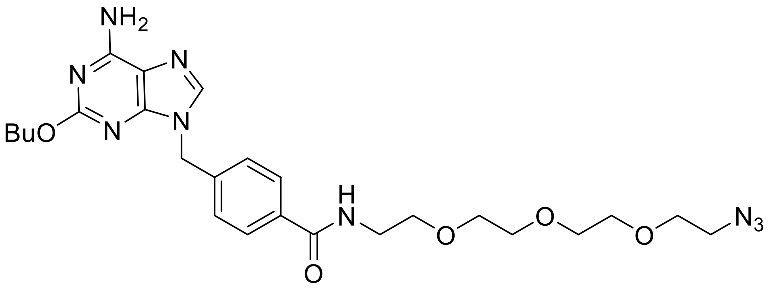

A mixture 2-butoxy-9H-purin-6-amine **17** (110 mg, 0.53 mmol) and K_2CO_3 (220 mg, 1.53 mmol) in anhydrous DMF (2 mL) was stirred under N_2 at 65ºC for 30 min. The azide derivative **16** (331 mg, 0.80 mmol) was added and the mixture was stirred for 72 h. The mixture was filtered, and the solvent was evaporated under vacuum. Then, the residue was diluted with chloroform and washed with distilled water, and dried over anhydrous magnesium sulfate. The organic layer was concentrated under reduced pressure and purified by column chromatography on silica gel (chloroform/methanol, 25:1) to afford the **17** as a colourless oil (199 mg, 69%). ^{1}H NMR ($CDCl_3$, 400 MHz) δ: 7.80 (d, *J* = 7.7 Hz, 2H, ArC$\underline{H}$), 7.62 (s, 1H, NC$\underline{H}$N), 7.37 (d, *J* = 7.7 Hz, 2H, ArC$\underline{H}$), 6.80 (brs, 1H, N$\underline{H}$), 5.56 (brs, 2H, N$\underline{H_2}$), 5.34 (s, 2H, NC$\underline{H_2}$), 4.34 (t, *J* = 6.5 Hz, 2H, $CH_3CH_2CH_2C\underline{H_2}$), 3.64-3.33 (m, 14H, overlapped with water signal, C$\underline{H_2}$PEG), 3.34 (m, 2H, $C\underline{H_2}N_3$), 1.80 (m, 2H, $CH_3CH_2C\underline{H_2}CH_2$), 1.52 (q, *J* = 7.5 Hz, 2H, $CH_3C\underline{H_2}CH_2CH_2$), 0.99 (t, *J* = 7.5 Hz, 3H, $C\underline{H_3}CH_2CH_2CH_2$). ^{13}C-NMR (100 MHz, $CDCl_3$) δ: 166.8, 162.4, 156.5, 151.9, 139.2, 138.6, 134.5, 127.7, 127.2, 115.5, 70.61, 70.57, 70.51, 70.23, 70.0, 69.7, 67.1, 50.6, 46.5, 39.8, 31.0, 19.2, 13.9. ESI-MS m/z calcd. For $C_{25}H_{35}O_5N_9$: 541.3 $[M]^+$; found: 542.4 $[M + H]^+$. ESI-HRMS m/z calcd. for $C_{25}H_{36}O_5N_9$: 542.2840; found: 542.2834.

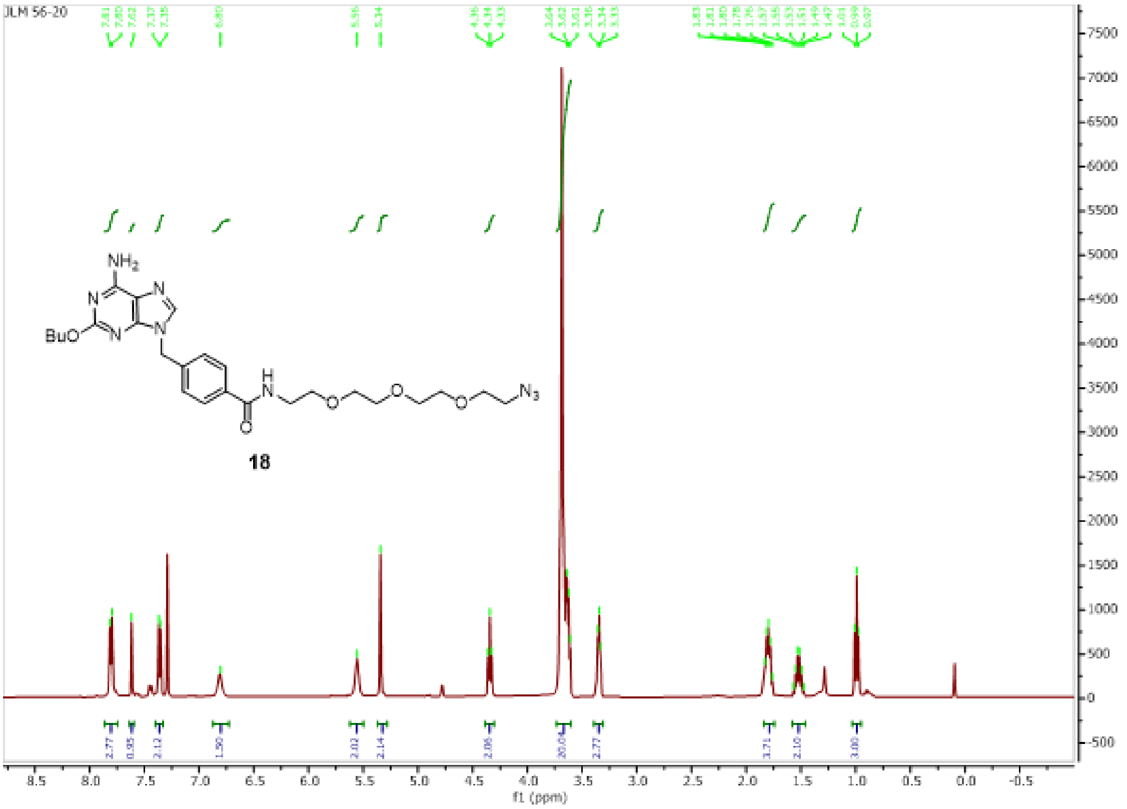

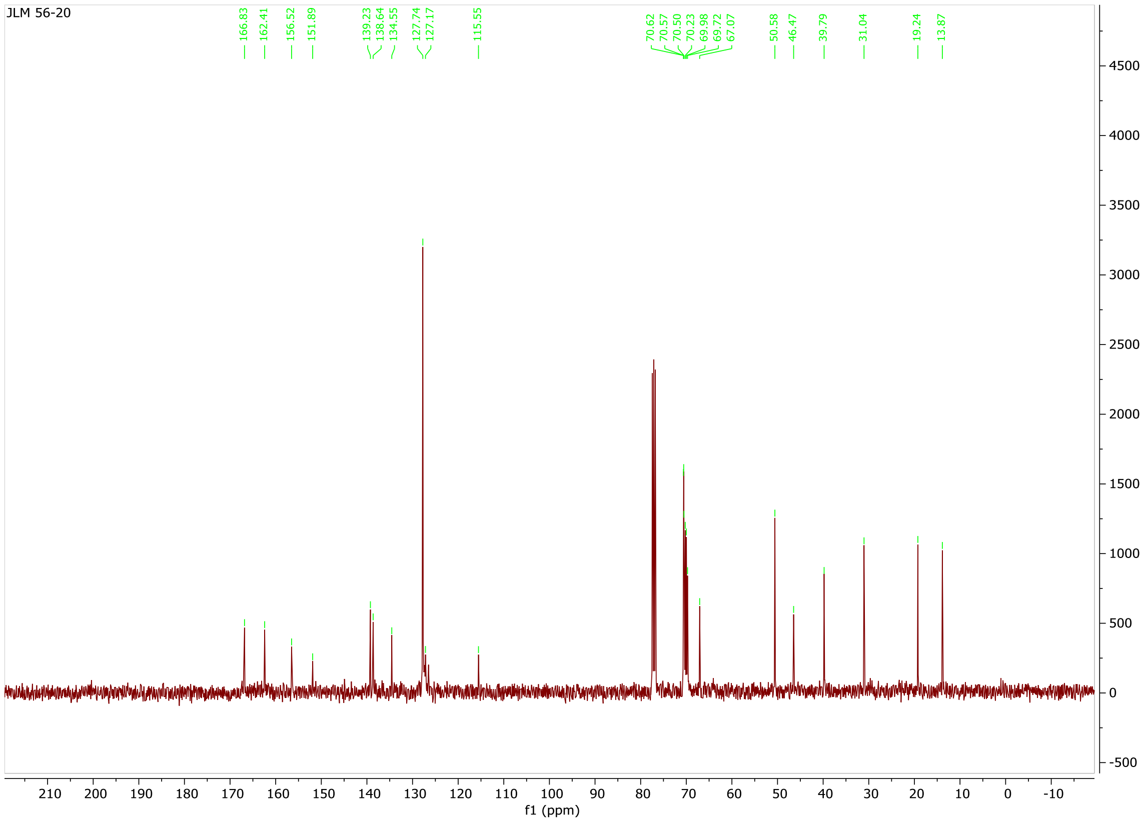

^{1}H-RMN (400 MHz, $CDCl_3$, 298 K) spectrum (up) and ^{13}C-RMN (100 MHz, $CDCl_3$, 298 K) spectrum (down) of compound **18**.

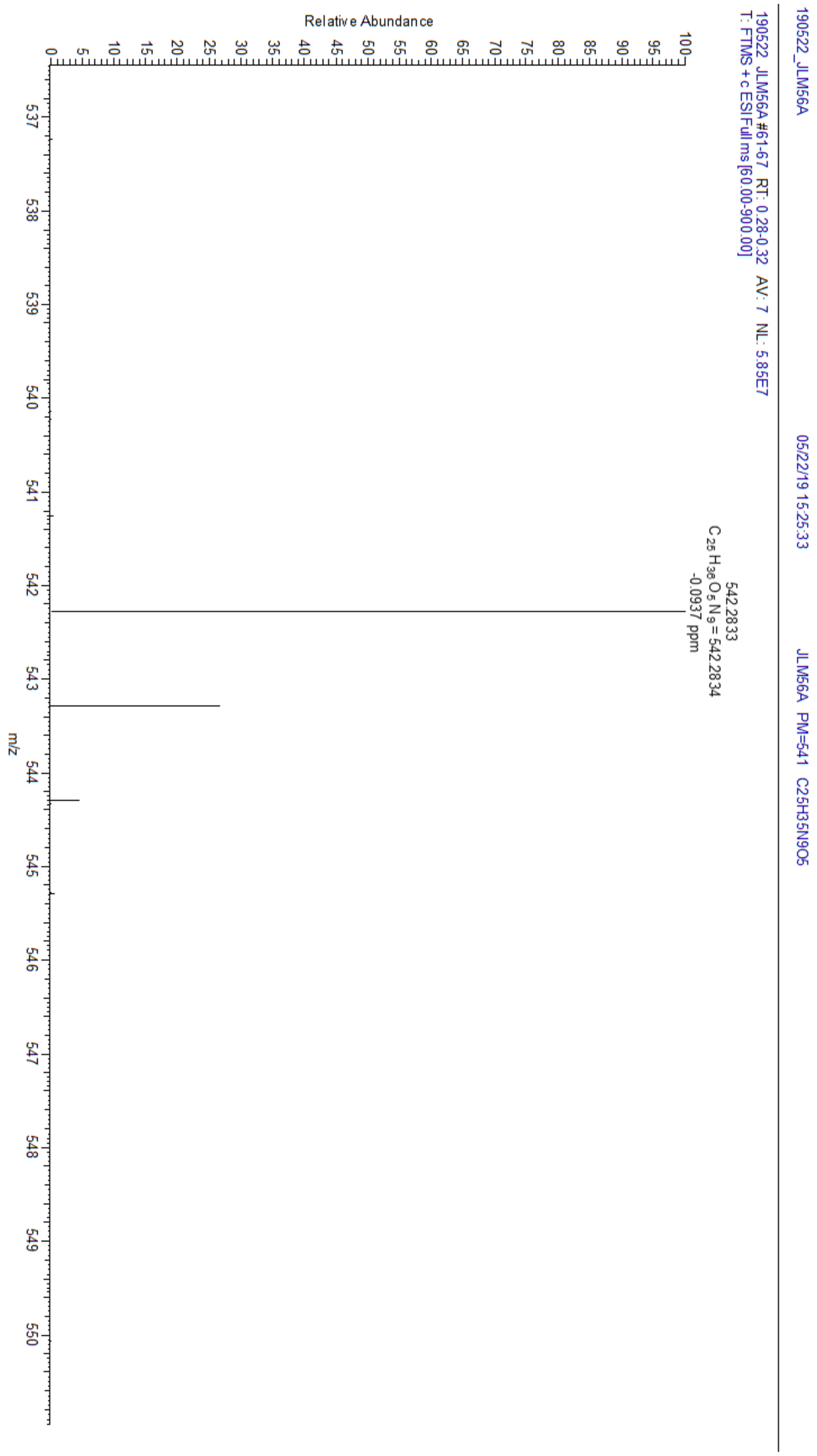

ESI-HRMS spectrum of compound **18**.

4-((6-amino-8-bromo-2-butoxy-7,8-dihydro-9H-purin-9-yl)methyl)-*N*-(2-(2-(2-(2-azidoethoxy)ethoxy)ethoxy)ethyl)benzamide (19)

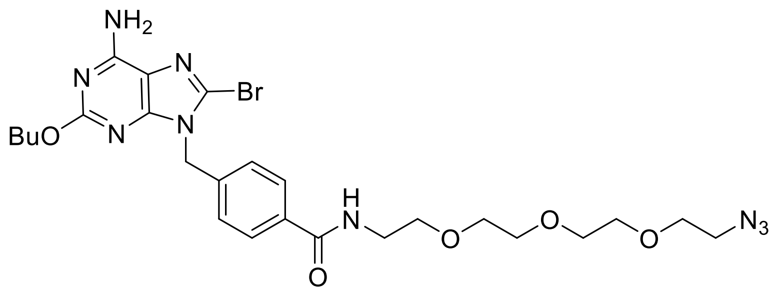

Adenine derivative **16** (609 mg, 1.12 mmol) was dissolved in chloroform (42 mL) and bromine (0.36 mL, 7 mmol) was added. The flask was fitted with a condenser and the resulting mixture was stirred at room temperature for 17 h in the dark. The flask was left open in the fumehood until most of the Br_2 was evaporated, and the solvent was removed under vacuum. The residue was purified by column chromatography on silica gel (chloroform/ methanol, 30/1) yielding **19** as a yellow oil (463 mg, 66%). ^{1}H NMR ($CDCl_3$, 400 MHz) δ: 7.78 (d, *J* = 7.7 Hz, 2H, ArC<u>H</u>), 7.37 (d, *J* = 7.7 Hz, 2H, ArC<u>H</u>), 6.93 (brs, 1H, N<u>H</u>), 5.92 (brs, 2H, N$\underline{H_2}$), 5.35 (s, 2H, NC$\underline{H_2}$), 4.31 (t, *J* = 6.6 Hz, 2H $CH_3CH_2CH_2C\underline{H_2}$), 3.67-3.33 (m, 14H, C$\underline{H_2}$PEG), 3.34 (m, 2H, C$\underline{H_2}N_3$), 1.79 (m, 2H, $CH_3CH_2C\underline{H_2}CH_2$), 1.48 (q, *J* = 7.5 Hz, 2H, $CH_3C\underline{H_2}CH_2CH_2$), 0.97 (t, *J* = 7.5 Hz, 3H, $C\underline{H_3}CH_2CH_2CH_2$). ^{13}C-NMR (100 MHz, $CDCl_3$) δ: 166.9, 162.2, 155.2, 153.0, 138.6, 134.5, 127.8, 127.6, 124.3, 116.0, 70.7, 70.6, 70.5, 70.3, 70.0, 69.8, 67.3, 50.6, 46.9, 39.8, 31.0, 19.2, 13.9. ESI-MS m/z calcd. For $C_{25}H_{34}O_9N_9Br$: 619.2 $[M]^+$; found: 642.2 $[M + Na]^+$. ESI-HRMS m/z calcd. for $C_{25}H_{35}O_5N_9Br$: 620.1945; found: 620.1929.

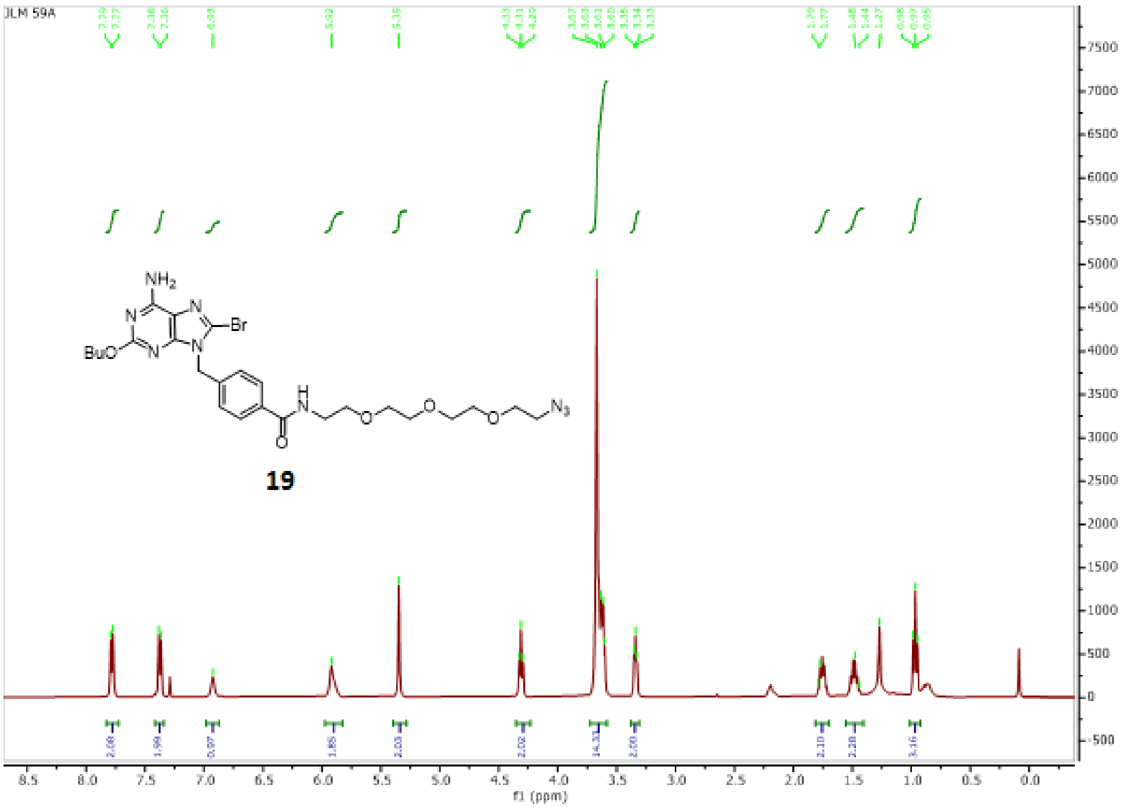

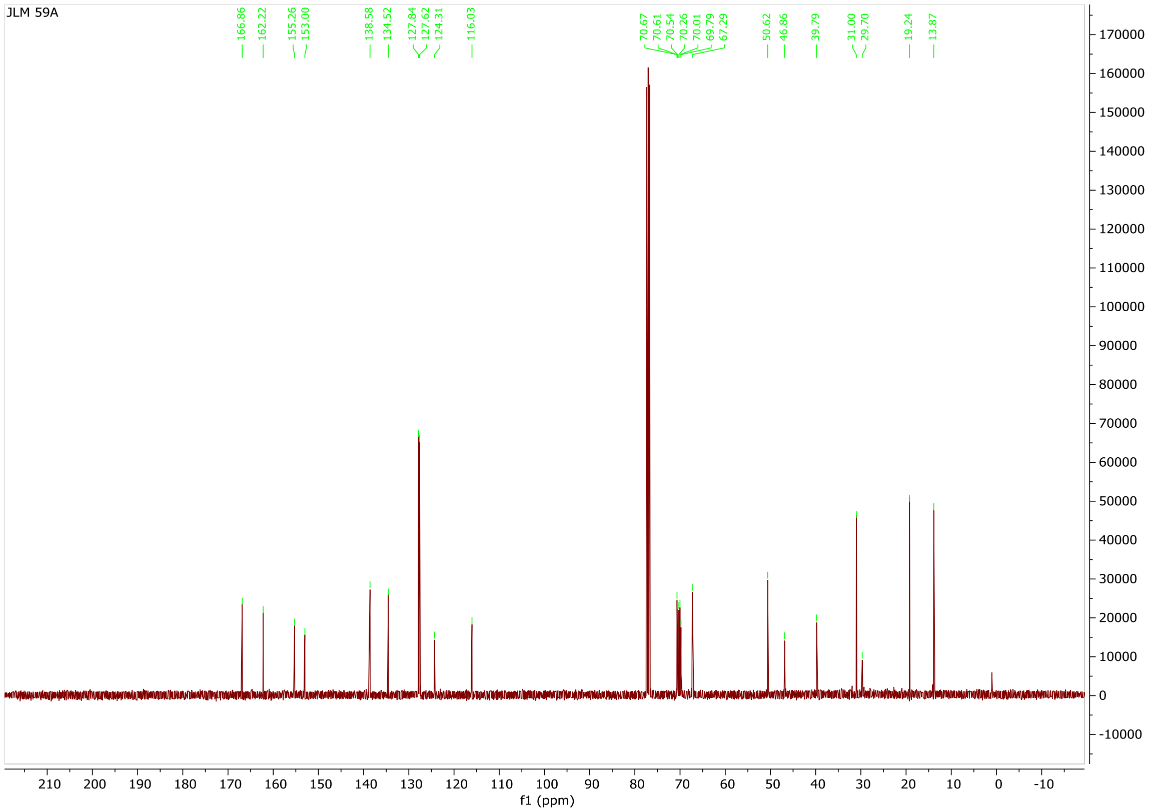

^{1}H-RMN (400 MHz, $CDCl_3$, 298 K) spectrum (up) and ^{13}C-RMN (100 MHz, $CDCl_3$, 298 K) spectrum (down) of compound **19**.

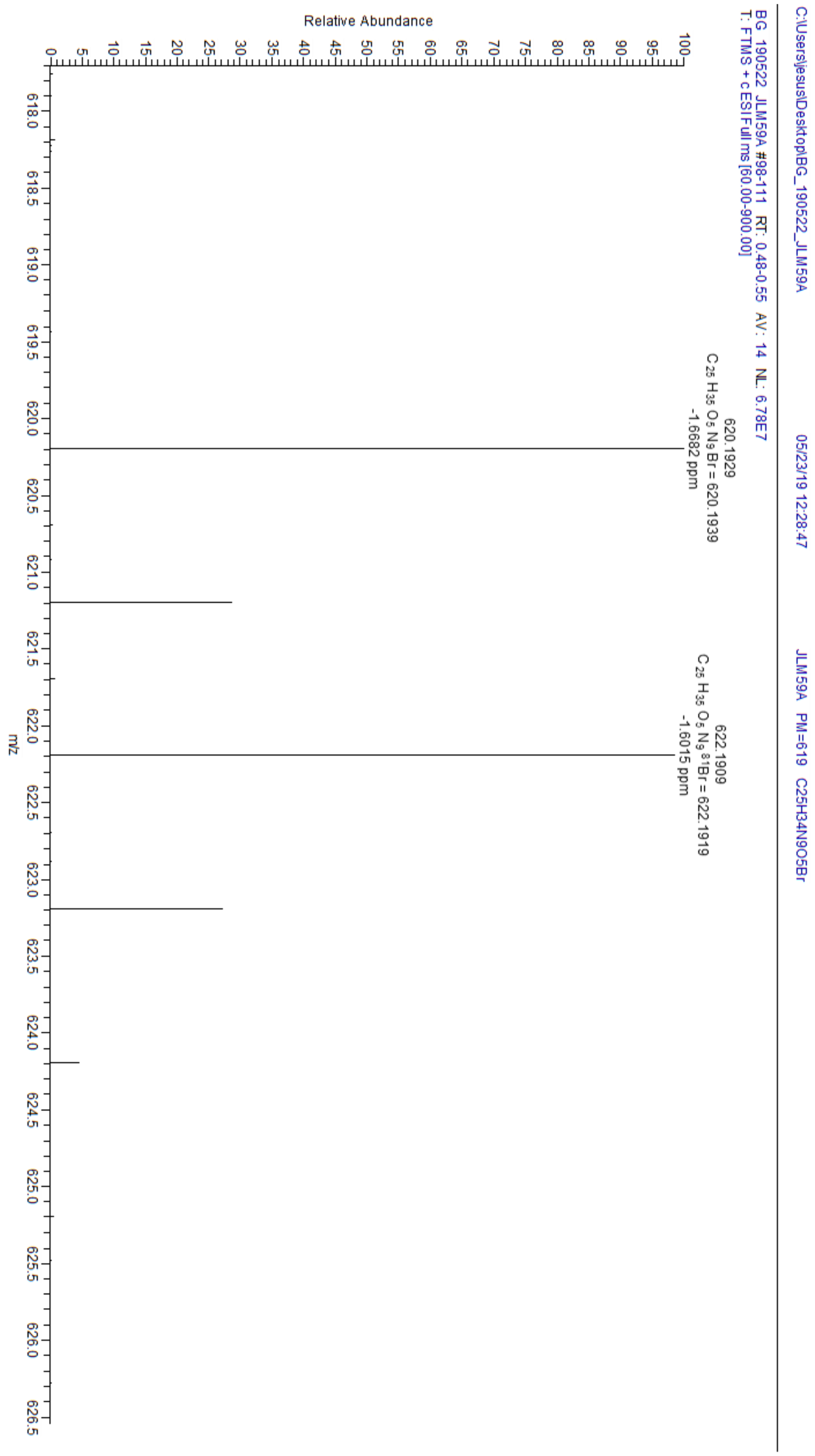

ESI-HRMS spectrum of compound **19**.

4-((6-amino-2-butoxy-8-oxo-7,8-dihydro-9H-purin-9-yl)methyl)-*N*-(2-(2-(2-(2-azidoethoxy)ethoxy)ethoxy)ethyl)benzamide (20)

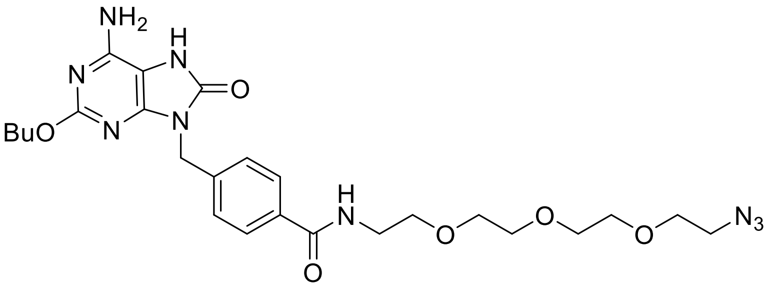

A mixture of 8-bromoadenine derivative **19** (463 mg, 0.747 mmol) in concentrated HCO_2H (45 mL) was stirred at reflux overnight. The HCO_2H was co-evaporated with distilled water (3x50 mL), and the residue was dried under vacuum. The residue was purified by column chromatography on silica gel (chloroform/ methanol, 20:1) to afford the **20** as a white amorphous solid (192 mg, 47%). ^1H NMR (DMSO-d_6, 400 MHz) δ: 10.0 (s, 1H, N<u>H</u>), 8.46 (brs, 1H, OCN<u>H</u>-PEG), 7.80 (d, *J* = 7.9 Hz, 2H, ArC<u>H</u>), 7.34 (d, *J* = 7.6 Hz, 2H, ArC<u>H</u>), 6.47 (brs, 2H, N<u>H₂</u>), 4.91 (s, 2H, NC<u>H₂</u>), 4.14 (t, *J* = 6.8 Hz, 2H, $CH_3CH_2CH_2C\underline{H_2}$), 3.58-3.33 (m, 16H, overlapped with water signal, $C\underline{H_2}$PEG), 1.61 (q, *J* = 7.3 Hz, 2H, $CH_3CH_2C\underline{H_2}CH_2$), 1.37 (q, *J* = 7.3 Hz, 2H, $CH_3C\underline{H_2}CH_2CH_2$), 0.91 (t, *J* = 7.3 Hz, 3H, $C\underline{H_3}CH_2CH_2CH_2$). ^{13}C-NMR (100 MHz, DMSO-d_6) δ: 166.4, 160.5, 152.7, 149.6, 148.2, 140.6, 134.0, 127.9, 98.7, 70.2, 70.1, 69.7, 69.3, 66.3, 55.4, 50.4, 42.6, 31.0, 19.2, 14.1. ESI-MS m/z calcd. For $C_{25}H_{23}O_6N_9$: 557.3 $[M]^+$; found: 580.2 $[M + Na]^+$. ESI-HRMS m/z calcd. for $C_{25}H_{36}O_6N_9$: 558.2789; found: 558.2274.

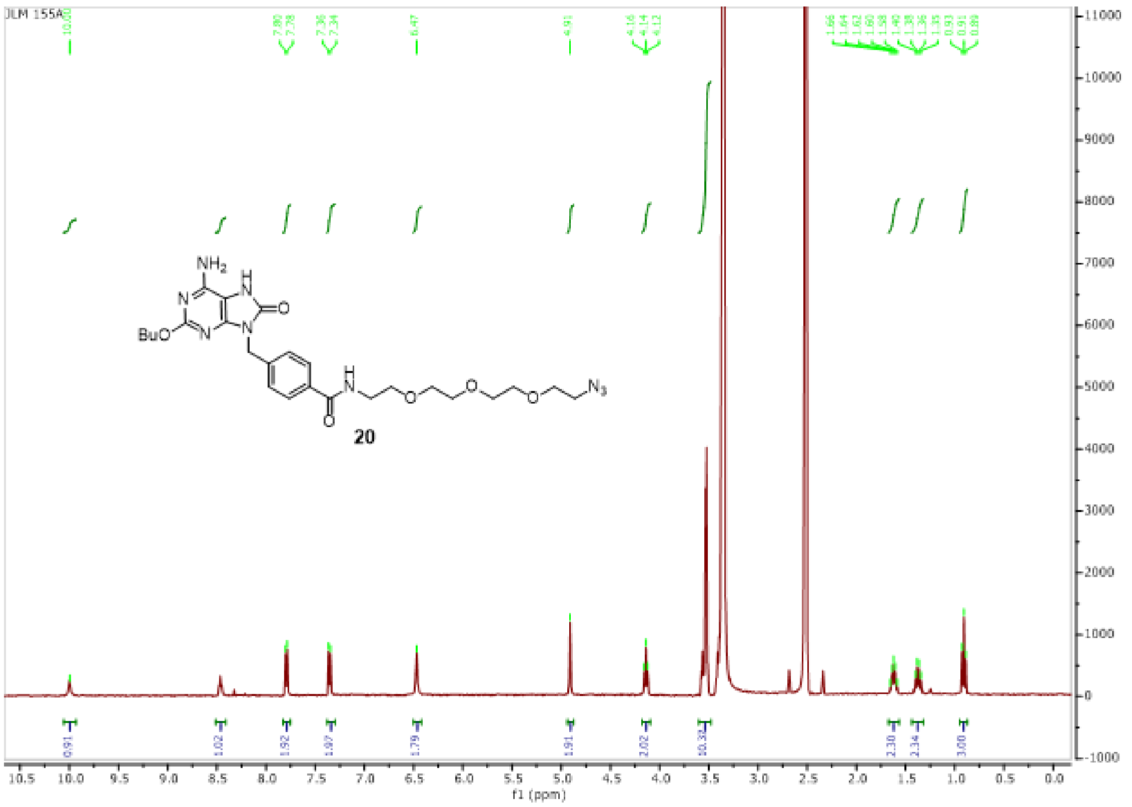

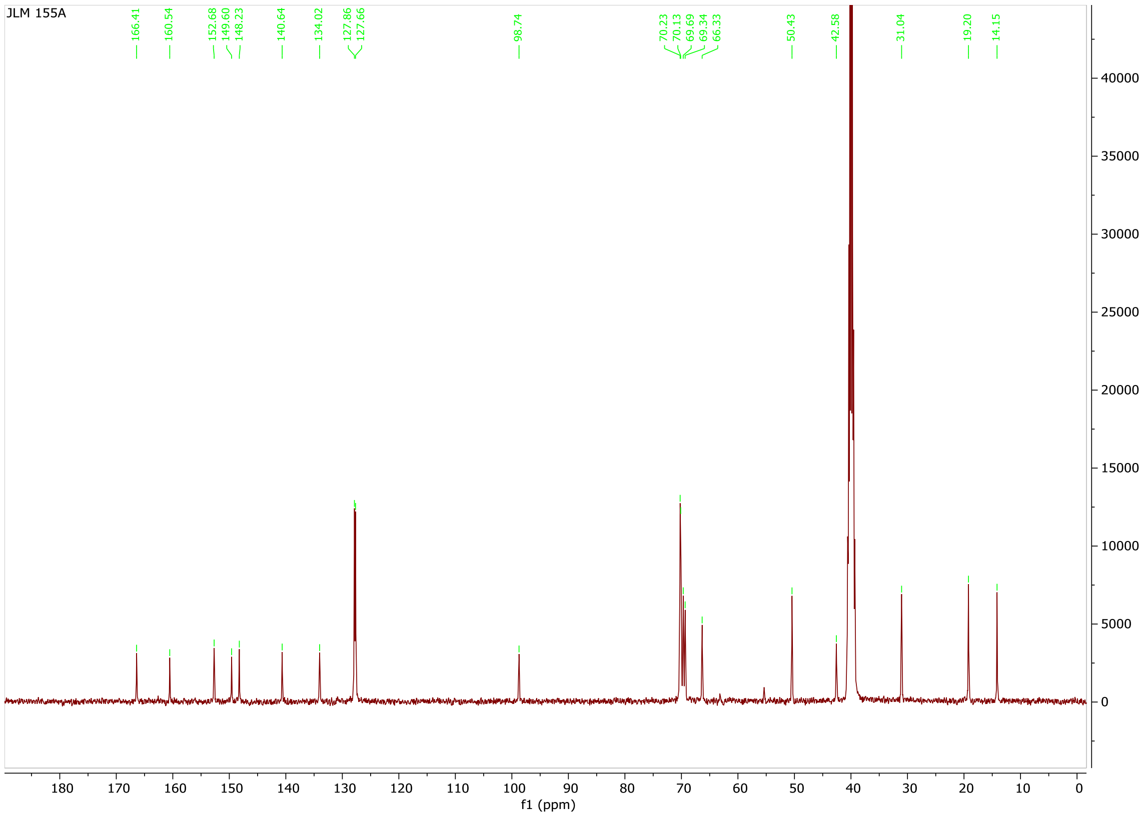

^{1}H-RMN (400 MHz, DMSO-d_6, 298 K) spectrum (up) and ^{13}C-RMN (100 MHz, DMSO-d_6, 298 K) spectrum (down) of compound **20**.

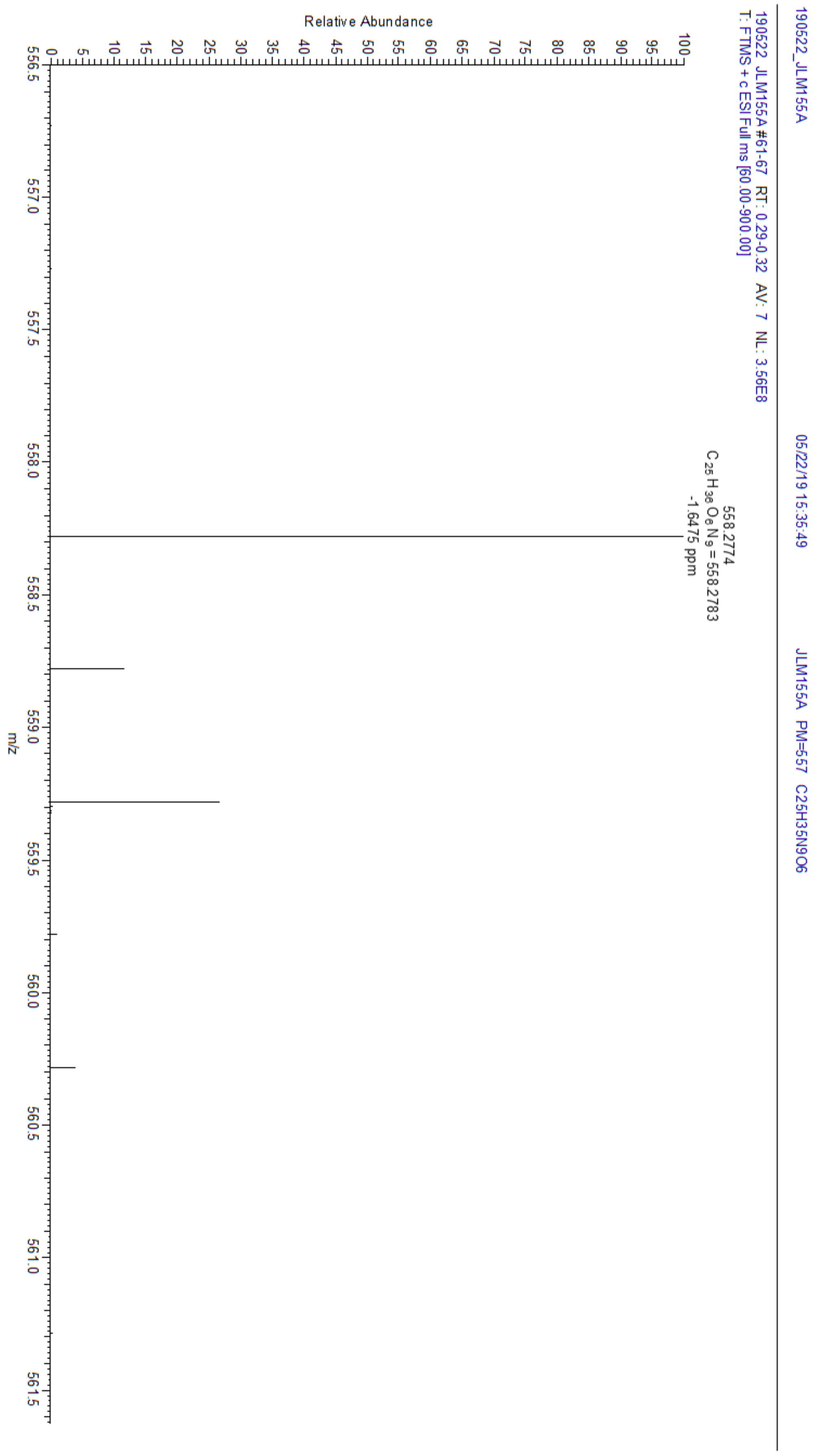

ESI-HRMS spectrum of compound **20**.

(1-(1-(4-((6-amino-2-butoxy-8-oxo-7,8-dihydro-9H-purin-9-yl)methyl)phenyl)-1-oxo-5,8,11-trioxa-2-azatridecan-13-yl)-1H-1,2,3-triazol-4-yl)methyl 3-(2,5-dioxo-2,5-dihydro-1H-pyrrol-1-yl)propanoate (21)

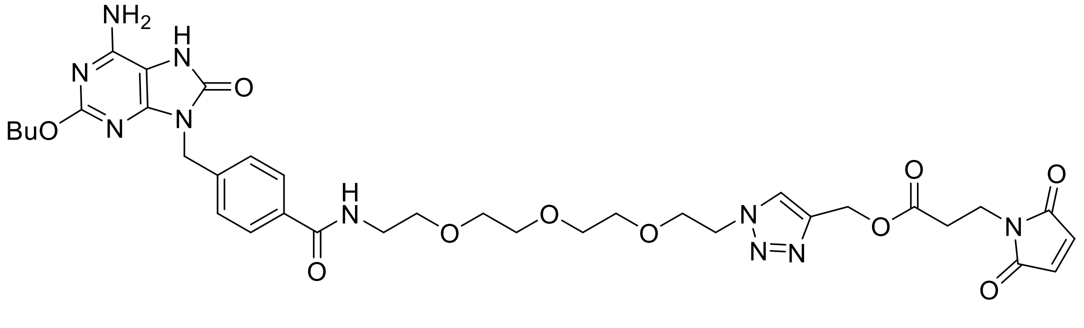

A solution of CuBr (1.55 mg, 0.01 mmol) in acetonitrile:DMSO (10:1, 0.6 mL) was added to a flask containing TentaGel™-TBTA resin (159 mg, 0.17 mmol/g, 0.03 mmol) under argon atmosphere. The mixture was stirred for 10 min in the dark. Then, a solution of azide **20** (20 mg, 0.04 mmol) in DMSO (0.5 mL) and prop-2-yn-1-yl-3-maleimidopropanoate,[9] (15 mg, 0.07 mmol) in acetonitrile:DMSO (10:1, 0.4 mL) were added. Under argon atmosphere and darkness, two additional portions of CuBr (1.5 mg, 0.01 mmol) in the same mixture of acetonitrile / DMSO (0.2 mL) were added every 2 hours. Finally, the sample was shaking overnight. Afterwards, the solvent was evaporated under high vacuum and the residue was purified by column chromatography on silica gel (chloroform/ methanol, 25:1) to afford **21** as a white amorphous solid (26 mg, 94%).^{1}H NMR (DMSO-d_6 , 400 MHz) δ: 9.99 (s, 1H, N<u>H</u>), 8.46 (brs, 1H, OCN<u>H</u>-PEG), 8.08 (s, 1H, $H_{triazole}$), 7.80 (d, *J* = 7.9 Hz, 2H, ArC<u>H</u>), 7.34 (d, *J* = 8.0 Hz, 2H, ArC<u>H</u>), 6.99 (s, 2H, OCC<u>H</u>), 6.47 (brs, 2H, N<u>H₂</u>), 5.10 (s, 2H, CC<u>H₂</u>OCO), 4.91 (s, 2H, NC<u>H₂</u>), 4.51 (m, 2H, $CH_2C\underline{H_2}N$), 4.14 (t, *J* = 6.6 Hz, 2H, $CH_3CH_2CH_2C\underline{H_2}$), 3.79 (t, *J* = 5.0 Hz, 2H, $CH_2C\underline{H_2}$triazole), 3.65 (t, *J* = 7.0 Hz, 2H, $C\underline{H_2}CH_2$triazole), 3.52-3.38 (m, 12H, overlap water signal, $C\underline{H_2}$PEG), 2.61 (t, *J* = 7.0 Hz, 2H, $C\underline{H_2}CH_2N$), 1.61 (q, *J* = 7.3 Hz, 2H, $CH_3CH_2C\underline{H_2}CH_2$), 1.38 (q, *J* = 7.3 Hz, 2H, $CH_3C\underline{H_2}CH_2CH_2$), 0.90 (t, *J* = 7.3 Hz, 3H, $C\underline{H_3}CH_2CH_2CH_2$). ^{13}C-NMR (100 MHz, DMSO-d_6) δ: 171.1, 170.7, 166.4, 160.6, 152.7, 149.6, 148.3, 141.9, 140.6, 135.0, 134.0, 127.9, 127.6, 125.6, 98.7, 70.14, 70.06, 69.97, 69.32, 69.06, 66.32, 57.9, 49.8, 42.6, 33.6, 32.7, 31.0, 19.2, 14.2. ESI-MS m/z calcd. For $C_{35}H_{44}O_{10}N_{10}$: 764.6 $[M]^+$; found: 787.2 $[M + Na]^+$. ESI-HRMS m/z calcd. for $C_{35}H_{45}O_{10}N_{10}$: 765.332; found:765.3304. RP-HPLC t_R = 11.07 min (Column: analytical BioZen, 3.6 µm, Peptide XB-C8; A: H_2O (0.05 % TFA), B: Acetonitrile (0.1 % TFA), 10 to 60% linear gradient of B into A over 15 min, F = 1 mL/min).

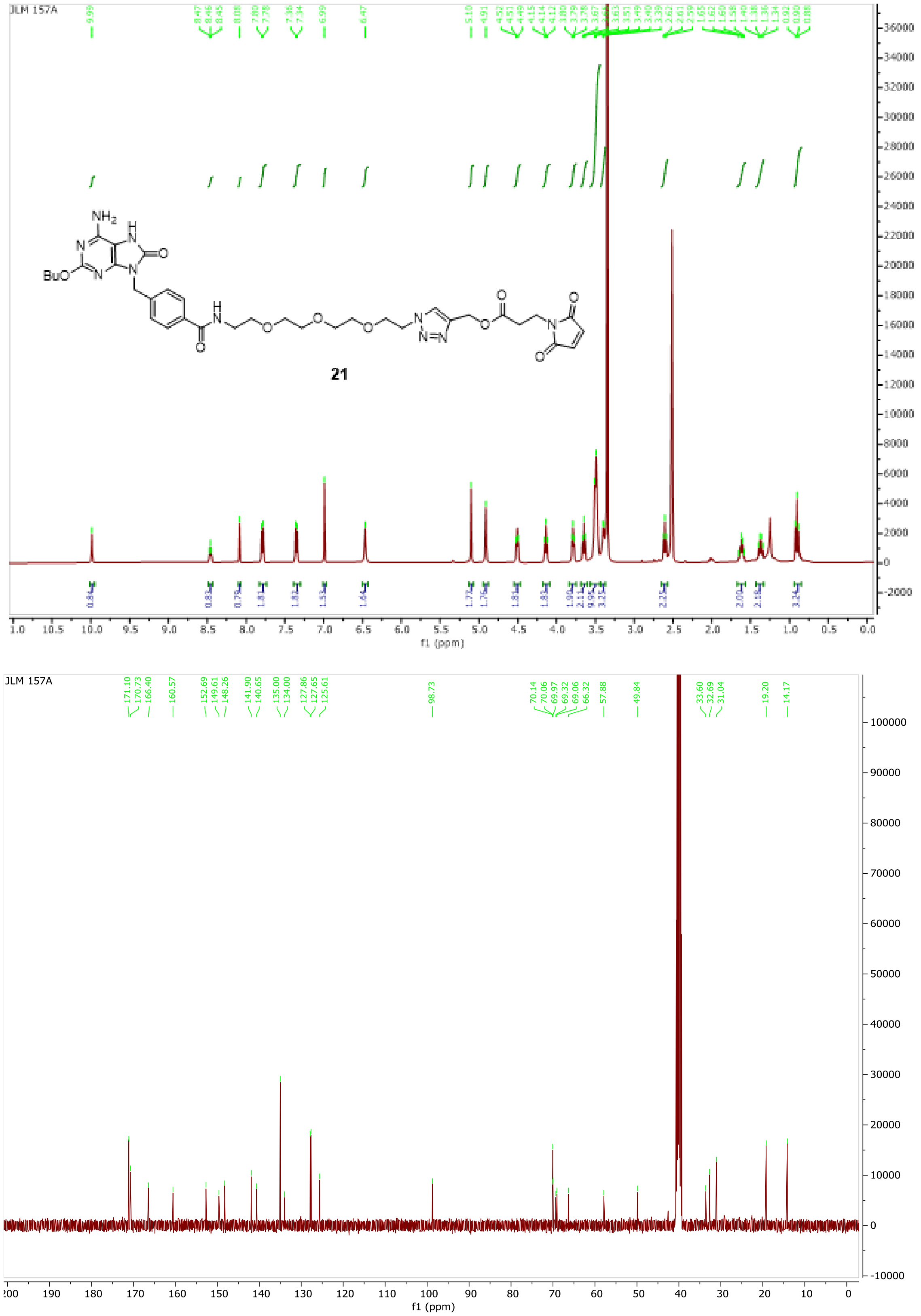

^{1}H-RMN (400 MHz, DMSO-d_6, 298 K) spectrum (up) and ^{13}C-RMN (100 MHz, DMSO-d_6, 298 K) spectrum (down) of compound **21**.

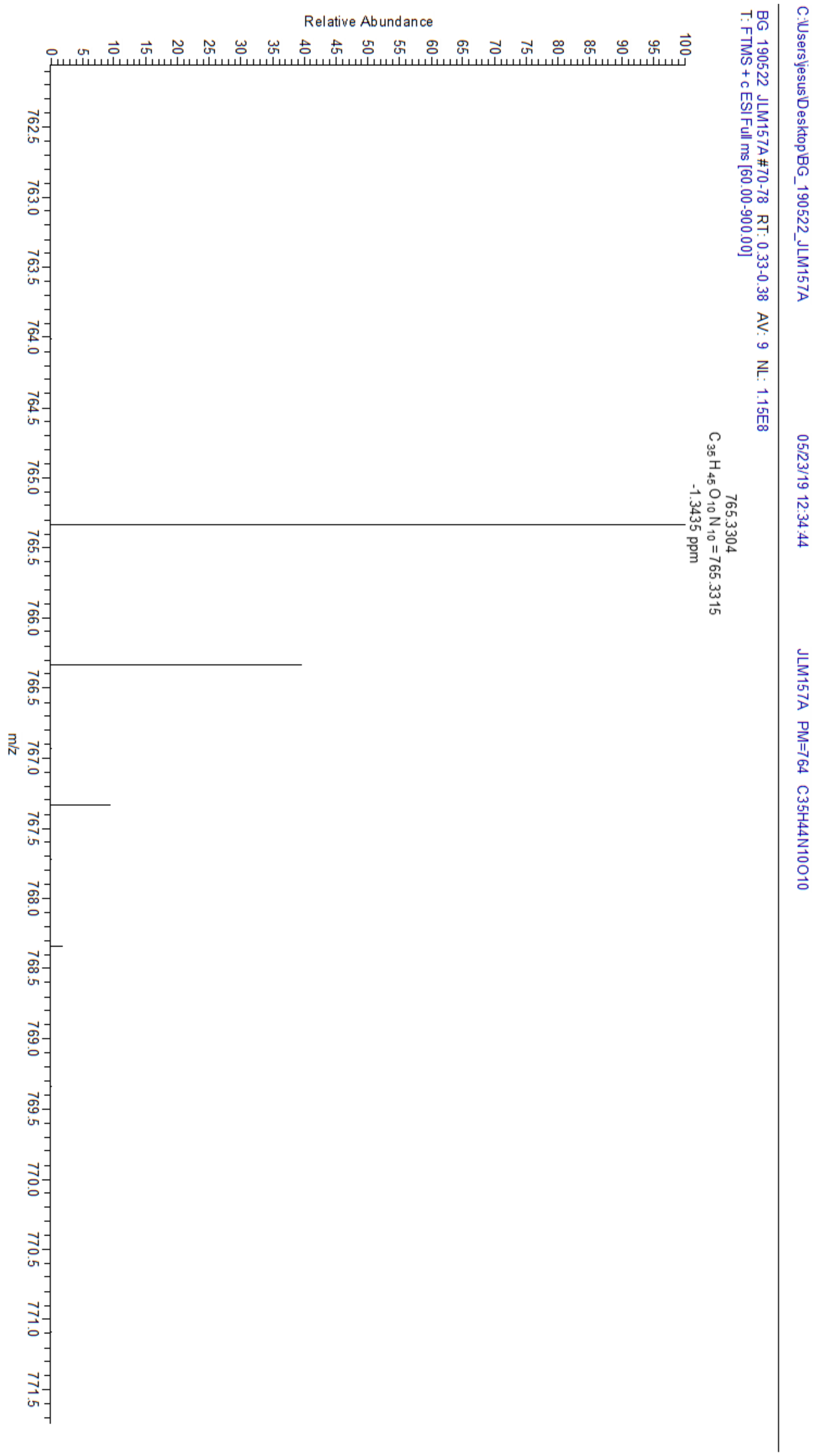

ESI-HRMS spectrum of compound **21**.

TLR7lig-Pp3 (2)

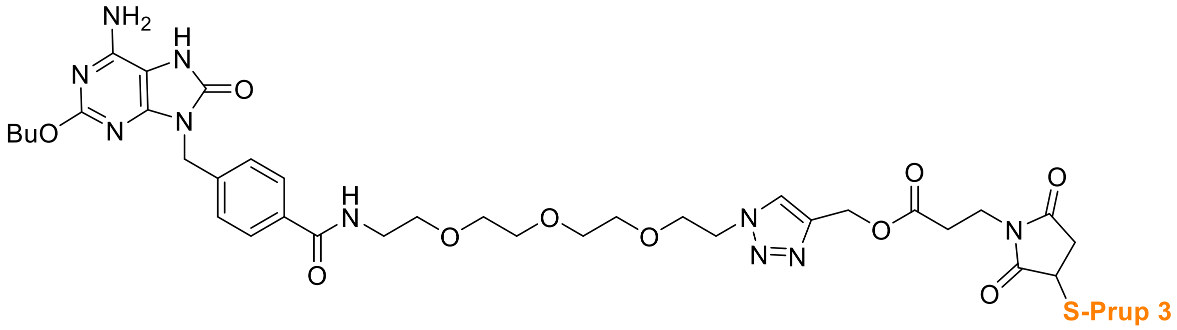

Following the general procedure, using maleimide derivative **21** (0.59 µmol) as starting material, conjugate **2** was obtained as a white solid (1.7mg, 96%). ESI-MS m/z calcd.: 3019.9 $[M]^{+}$; found: 1511.0 $[M + 2H]^{+2}$, 1007.9 $[M + 3H]^{+3}$, 756.3 $[M + 4H]^{+4}$,605.3 $[M + 5H]^{+5}$. RP-HPLC t_R = 8.91 min (Column: analytical BioZen, 3.6 µm, Peptide XB-C8; A: H_2O (0.05 % TFA), B: Acetonitrile (0.1 % TFA), 10 to 60% linear gradient of B into A over 15 min, F = 1 mL/min).

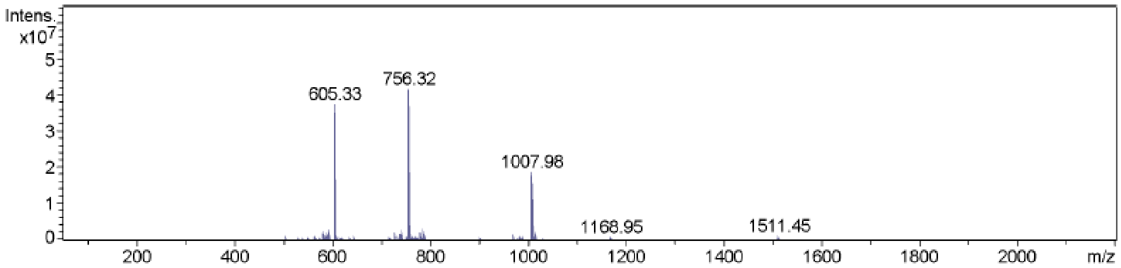

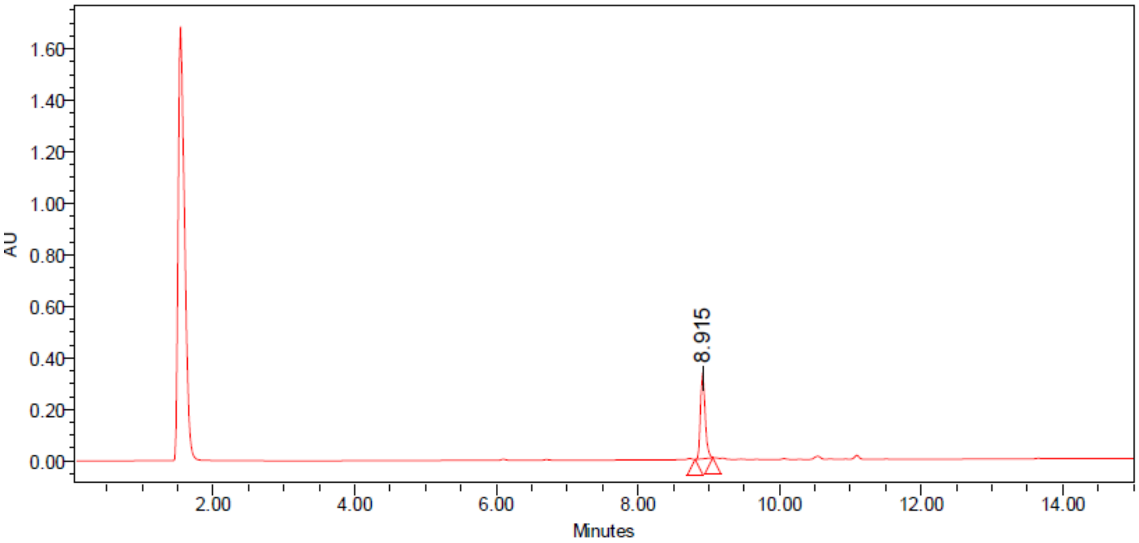

3. Figure S1

Figure S1

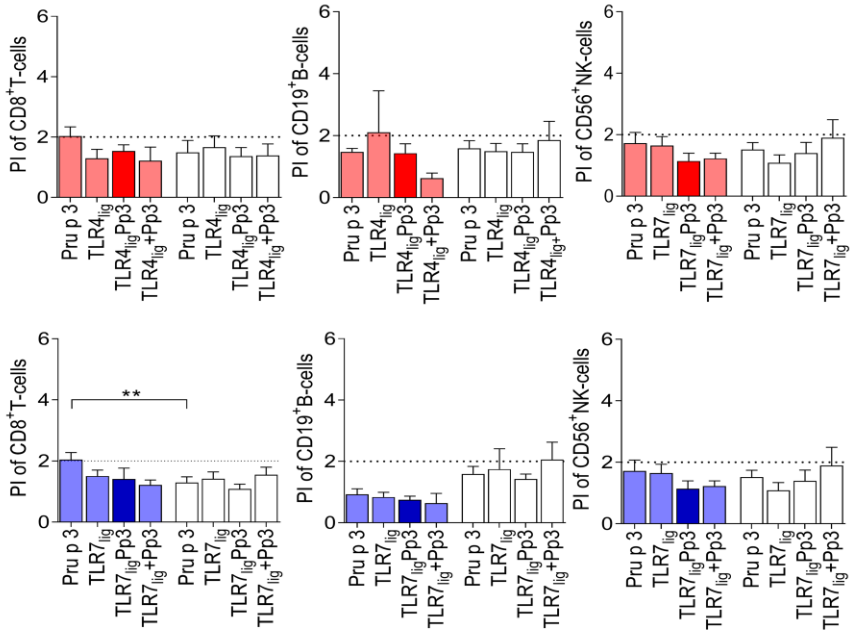

Figure S1. Specific lymphocyte proliferation in the presence of both TLRn$_{lig}$-Pp3. Bars represent median and SEM of the proliferation index (PI) of T- (CD8$^+$) B-(CD19$^+$) and NK (CD56$^+$)-cells for Pp3, TRLn$_{lig}$, TRLn$_{lig}$-Pp3 and TRLn$_{lig}$ plus Pp3 for allergic patients (n = 9) and tolerant controls (n = 9) at 10 nM. Mann–Whitney U test was used for pairwise comparisons between unrelated groups, showing significant differences as **, ($p < 0.0125$ Bonferroni correction). The dotted line represents the PI > 2.

4. Figure S2

Figure S2

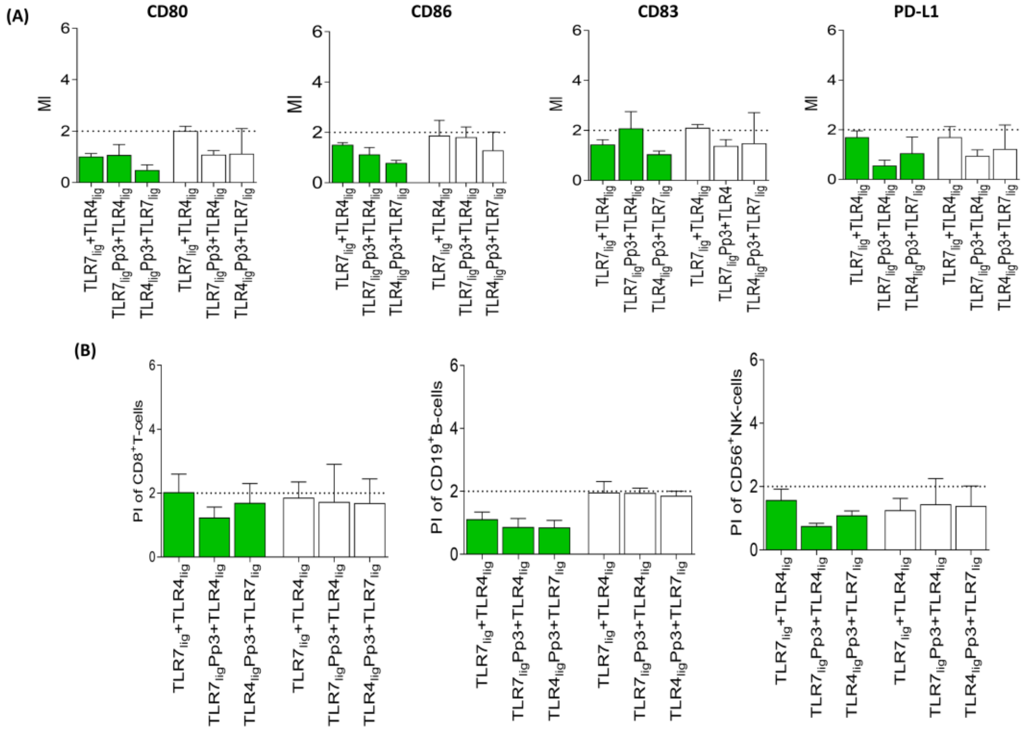

Figure S2. The combination of $TRLn_{lig}$ with or without Pp3 do not change moDC maturation and nor does induce the proliferation of different cell populations. Bars represent median and SEM of the (A) maturation index (MI) for the different surface markers on moDC, and (B) the proliferation index (PI) of T- ($CD8^+$) B-($CD19^+$) and NK ($CD56^+$)-cells for the combination of $TRLn_{lig}$ with or without Pp3 for allergic patients (n = 9) and tolerant controls (n = 9) at 10 nM. The dotted line represents the MI and PI > 2, respectively.

5. Table S1

Table S1. Clinical characteristics

N. Subjects	Sex	Age	Type reaction	SPT LTP	sIgE Pru p 3
**P1**	F	49	Anaphylaxis	+	13.30
**P2**	F	22	OAS	+	13,20
**P3**	M	29	OAS	-	0.62
**P4**	F	49	Contact urticaria	+	41.9
**P5**	M	23	Contact urticaria	+	0.35
**P6**	F	34	Anaphylaxis	+	8.73
**P7**	F	44	Angioedema	+	80.8
**P8**	F	31	OAS	+	3.37
**P9**	M	26	OAS	+	5.03
**C1**	M	49	Tolerant	-	0.35
**C2**	F	36	Tolerant	-	0.35
**C3**	F	30	Tolerant	-	0.35
**C4**	F	28	Tolerant	-	0.35
**C5**	M	50	Tolerant	-	0.35
**C6**	M	29	Tolerant	-	0.35
**C7**	F	28	Tolerant	-	0.35
**C8**	F	38	Tolerant	-	0.35
**C9**	M	27	Tolerant	-	0.35

OAS: Oral allergy syndrome.

References

1. Zysman-Colman, E.; Arias. K.; Siegel, J. S.; Synthesis of arylbromides from arenes and N-bromosuccinimide (NBS) in acetonitrile — A convenient method for aromatic bromination. *Can. J. Chem*. **2009**, *87*, 440- 447.
2. Chan, M.; Chan, M.; Kakitsubata, Y.; Hayashi, T.; Ahmadi, A.; Yao, S.; Shukla, N.M.; Oyama, S.-Y.; Baba, A.; Nguyen, B.; Corr, M.; *et al.* Structure-Activity Relationship Studies of Pyrimido[5,4-*b*]indoles as Selective Toll-Like Receptor 4 Ligands. *J. Med. Chem*. **2017**, *60*, 9142-9161.
3. Bakleh, M.E.; Sol V.; Estieu-Gionnet, K.; Granet, R.; Déléris, G.; Krausz, P. An efficient route to VEGF-like peptide porphyrin conjugates via microwave-assisted ‘click-chemistry. *Tetrahedron* **2009**, *65*, 7385-7392.
